# ASPsiRNA: A Resource of ASP-siRNAs Having Therapeutic Potential for Human Genetic Disorders and Algorithm for Prediction of Their Inhibitory Efficacy

**DOI:** 10.1534/g3.117.044024

**Published:** 2017-07-06

**Authors:** Isha Monga, Abid Qureshi, Nishant Thakur, Amit Kumar Gupta, Manoj Kumar

**Affiliations:** Bioinformatics Centre, Institute of Microbial Technology, Council of Scientific and Industrial Research, Chandigarh-160036, India

**Keywords:** allele-specific siRNA, ASPsiDb, ASPsiPred, genetic disease database, prediction algorithm

## Abstract

Allele-specific siRNAs (ASP-siRNAs) have emerged as promising therapeutic molecules owing to their selectivity to inhibit the mutant allele or associated single-nucleotide polymorphisms (SNPs) sparing the expression of the wild-type counterpart. Thus, a dedicated bioinformatics platform encompassing updated ASP-siRNAs and an algorithm for the prediction of their inhibitory efficacy will be helpful in tackling currently intractable genetic disorders. In the present study, we have developed the ASPsiRNA resource (http://crdd.osdd.net/servers/aspsirna/) covering three components *viz* (i) *ASPsiDb*, (ii) *ASPsiPred*, and (iii) analysis tools like *ASP-siOffTar*. ASPsiDb is a manually curated database harboring 4543 (including 422 chemically modified) ASP-siRNAs targeting 78 unique genes involved in 51 different diseases. It furnishes comprehensive information from experimental studies on ASP-siRNAs along with multidimensional genetic and clinical information for numerous mutations. ASPsiPred is a two-layered algorithm to predict efficacy of ASP-siRNAs for fully complementary mutant (Eff^mut^) and wild-type allele (Eff^wild^) with one mismatch by *ASPsiPred^SVM^* and *ASPsiPred^matrix^*, respectively. In ASPsiPred^SVM^, 922 unique ASP-siRNAs with experimentally validated quantitative Eff^mut^ were used. During 10-fold cross-validation (10nCV) employing various sequence features on the training/testing dataset (T737), the best predictive model achieved a maximum Pearson’s correlation coefficient (PCC) of 0.71. Further, the accuracy of the classifier to predict Eff^mut^ against novel genes was assessed by leave one target out cross-validation approach (LOTOCV). ASPsiPred^matrix^ was constructed from rule-based studies describing the effect of single siRNA:mRNA mismatches on the efficacy at 19 different locations of siRNA. Thus, *ASPsiRNA* encompasses the first database, prediction algorithm, and off-target analysis tool that is expected to accelerate research in the field of RNAi-based therapeutics for human genetic diseases.

RNA interference (RNAi) is an evolutionarily conserved phenomenon to inhibit gene expression in eukaryotes including mammals (Fire *et al.* 1998; Paulson and Gonzalez-Alegre 2006). One of the most important implications of RNAi technology is the development of potent and highly effective siRNAs imparting exquisite specificity (Keiser *et al.* 2015). They have already been utilized as a vital research tool for loss-of-function studies and the suppression of phenotypes generated by dominantly acting mutant genes (Rodriguez-Lebron and Paulson 2006). Thus, siRNA-mediated selective suppression of dominantly inherited mRNA transcripts holds curative potential for gain-of-function human genetic diseases (Lopes *et al.* 2016; Loy *et al.* 2012).

In this context, allele-specific RNAi (ASP-RNAi) is an innovative category of RNAi with the objective of suppressing the dominant mutant allele while sparing expression of the corresponding normal allele with the specificity of single-nucleotide differences between the two (Gonzalez-Alegre 2007). Therefore, allele-specific siRNAs (ASP-siRNAs) are potentially a novel and better remedial alternative for the treatment of autosomal dominant genetic disorders especially in cases where wild-type allele expression is crucial for organism survival (Miller *et al.* 2003). The mechanism of ASP-RNAi gene silencing is illustrated in Figure 1.

**Figure 1:**
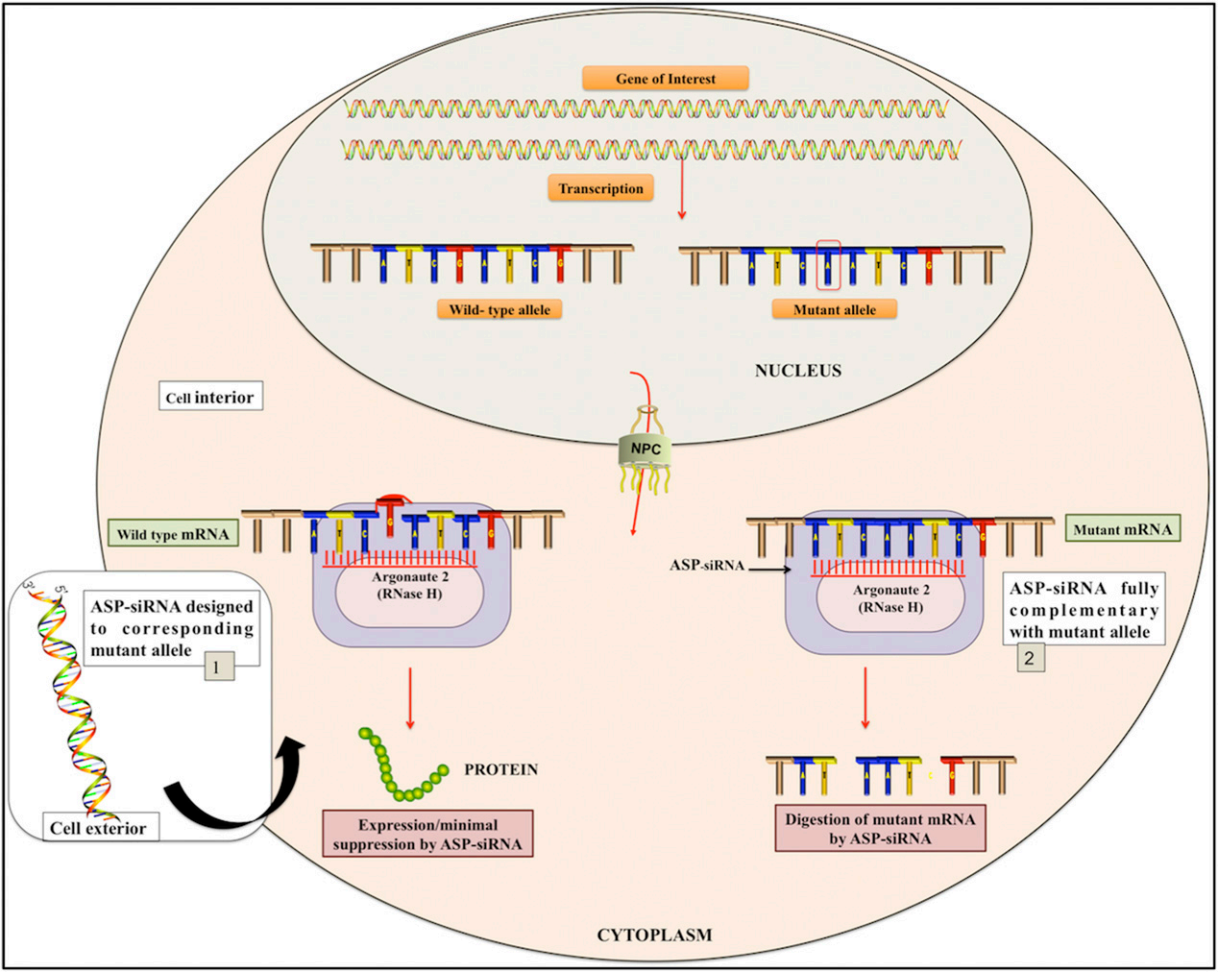
Mechanistic representation of ASP-RNAi.

Numerous studies have been conducted to assess the potency and specificity of ASP-siRNAs for various neurodegenerative disorders like Huntington disease (HD) (Drouet *et al.* 2014; Miniarikova *et al.* 2016), DYT1 dystonia (Gonzalez-Alegre *et al.* 2003, 2005), Alzheimer’s disease (Sierant *et al.* 2011), Parkinson’s disease (PD) (Takahashi *et al.* 2015), amyloid lateral sclerosis (ALS) (Schwarz *et al.* 2006), and Machado–Joseph disease (Alves *et al.* 2008). Their therapeutic potential has also been assessed for various skin disorders like epidermolysis bullosa simplex (Atkinson *et al.* 2011), epidermolytic palmoplantar keratoderma (EPPK) (Lyu *et al.* 2016), and lattice corneal dystrophy type I (LCDI) (Courtney *et al.* 2014). They have also been utilized to suppress the mutations associated with other diseases like cancer (Iyer *et al.* 2016), viral diseases (Teng *et al.* 2011), and sex-linked disorders (Caplen *et al.* 2002). Various *in-vivo* studies have been reported in different animal models, for *e.g.*, HD (Drouet *et al.* 2014), EPPK (Miniarikova *et al.* 2016), and hyper-trophic cardiomyopathy (Miniarikova *et al.* 2016). The potential of this therapeutic modality has been studied in human embryonic stem cells (Miniarikova *et al.* 2016), and allele-specific gene silencing (ASGS) approaches have started to move from the laboratory to the clinic (Liu *et al.* 2016). The first ASP-siRNA TD101 for the human skin disorder pachyonychia congenita (PC) has entered into phase1b clinical trials (Leachman *et al.* 2008).

Currently there is no cure available for dominant negative genetic maladies (Squitieri and de Yebenes 2015). Although, a few symptomatic pharmacological and nonpharmacological drugs have been used in clinical practice (Marelli and Maschat 2016), they were aimed at temporary relief and delay of disease progression (Jamwal and Kumar 2015; Kulshreshtha and Piplani 2016; LeWitt *et al.* 2016). Similarly peptide-based drugs have been used to suppress the aggregate formation of toxic mutant protein (Aharony *et al.* 2015; Arribat *et al.* 2013). However, it is reported that indiscriminate sustained suppression at the protein level may have harmful effects on the cell (Rodriguez-Lebron and Paulson 2006), and they are not aimed at disease reversal.

Likewise, traditional antisense molecules are also candidates for mutant-specific suppression (Pandey *et al.* 2015). However, the one-to-one ratio of binding to target requires high concentrations of these molecules in the cell, which may result in toxic situations (Allen *et al.* 2013). On the other hand, ASP-siRNAs exhibit multiplicity *i.e.*, a single siRNA can cause cleavage of multiple copies of the target mRNA (Allen *et al.* 2013). Moreover, antisense molecules exhibit irreversible binding to their target making them poor candidates for ASP-RNAi, especially when the system demands one nucleotide discrimination (Allen *et al.* 2013). Antisense Oligonucleotide (ASO), being single stranded, is unstable and less potent, thus requiring high concentrations and, consequently, leading to off-target effects more severe than dsRNA (Watts and Corey 2012).

Despite unprecedented specificity and immense therapeutic utility of ASP-siRNAs, bioinformatics repositories in the field are lacking. Although there are several resources available for siRNAs like siRECORDS (Ren *et al.* 2006), HusiDa (Truss *et al.* 2005), HIVsirDB (Tyagi *et al.* 2011), VIRsiRNAdb (Thakur *et al.* 2012b), siRNAmod (Dar *et al.* 2016b), and RNAiAtlas (Mazur *et al.* 2012), they lack information related to ASP-siRNAs (Supplemental Material, Table S1 in File S1). Likewise, there are numerous algorithms (Ahmed and Raghava 2011; Dar *et al.* 2016a; Filhol *et al.* 2012; Huesken *et al.* 2005; Kaur *et al.* 2016; McQuisten and Peek 2009; Mysara *et al.* 2011; Pan *et al.* 2011; Peek 2007; Qureshi *et al.* 2013; Saetrom 2004; Shabalina *et al.* 2006; Vert *et al.* 2006) and design rules (Amarzguioui and Prydz 2004; Elbashir *et al.* 2001a; Reynolds *et al.* 2004; Ui-Tei *et al.* 2004) for siRNA efficacy prediction. But, none of the available web servers was dedicated to predicting two efficacies associated with a single siRNA.

This prompted us to develop *ASPsiRNA*, a web resource offering multiple modules. The first module, ASPsiDb, delivers updated and manually curated ASP-siRNA sequences targeted against human genetic diseases available in the literature, coupled with clinicopathogenic information about various mutations and the annotation of genes. In the second module *ASPsiPred*, using data from the database, we have developed a two-layered algorithm for prediction of inhibitory efficacy of ASP-siRNA for mutant and wild-type alleles. We have provided Support Vector Machine (SVM) and matrix-based algorithms for the prediction of the efficacy of ASP-siRNA for both diseased (Eff^mut^) and wild-type alleles (Eff^wild^). This algorithm is aimed to help experimental biologists in designing optimum allele discriminatory siRNAs along with minimum off-targets. In the third module, we have integrated useful analysis tools like *ASP-siOffTar* (seed and full sequence based), *BLAST*, and *ASP-siMAP*.

## Materials and Methods

### ASPsiDb database development

#### Data collection:

Information extraction was primarily divided into four parallel data systems (Supplemental Methods Section I and II in File S1): (a) ASP-siRNA data extraction: An extensive literature search was executed to obtain articles indexed in PubMed using the following combination of keywords (((Allele)) AND (((((((sirna) OR shrna) OR small interfering RNA) OR short interfering RNA) OR RNA interference) OR RNAi) OR silencing)) AND (((specific) OR mismatch*) OR discrimination). Patents pertaining to ASP-siRNAs were extracted from “The Lens” (www.lens.org). (b) Clinical information regarding various mutations: Clinical data associated with different mutations were mined from ClinVar (Landrum *et al.* 2014), dbVar (Lappalainen *et al.* 2013), dbSNP (Sherry *et al.* 1999), and OMIM (Hamosh *et al.* 2000). (c) Annotation of genes targeted by ASP-siRNAs: It involves standard nomenclature of every gene from HGNC (HUGO Gene Nomenclature Committee), cytogenic/chromosomal coordinates of a gene from UniProt, UCSC genome browser. (d) Molecular/biological/genetic information regarding diverse human genes and corresponding diseases: Information about the genetic basis of disorders was compiled from various resources; *e.g.*, OMIM, ClinVar, and KEGG disease modules.

#### Database schema:

**Figure 2:**
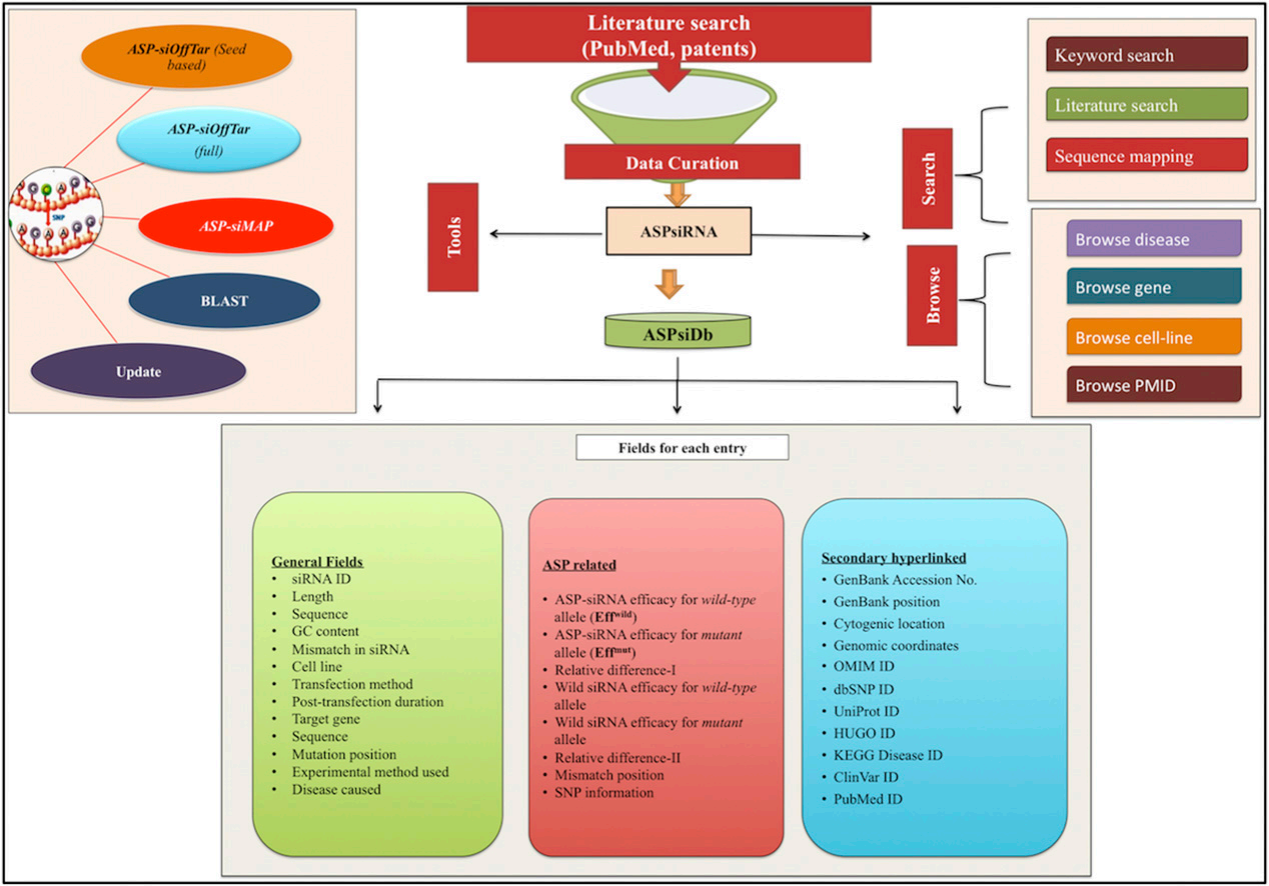
ASPsiRNA architecture.

Database content is systematically organized to provide easy access of ASP-siRNAs data coupled with comprehensive information of clinical and genetic data. It is maintained using MySQL and launched on Apache HTTP Server installed on an IBM machine under Red Hat Enterprise Linux5 background. The responsive front end was implemented with CSS, PHP, HTML5, and JavaScript as employed in our previous resources (Qureshi *et al.* 2014). Detailed architecture of the resource is depicted in Figure 2.

#### ASPsiDb web interface: searching and browsing:

Proficient searching and browsing is provided in the resource “Search” section that provides three suboptions for convenient data mining in the database, *i.e.*, (i) *keyword search*, (ii) *literature search*, and (iii) *sequence mapping* based search (Figure S1 in File S1). Additionally, we have also offered database browsing in six categories: disease, gene, mutation, cell line, mismatch, and Pubmed ID (Supplemental Methods Section III in File S1).

The output of the searching and browsing page provides a list of ASP-siRNAs matching the input query. By clicking on the individual ASP-siRNA ID, the user can get complete details of the respective entry structured in nine modules (Supplemental Methods Section IV and Figures S2–S5 in File S1).

### ASPsiPred: prediction algorithm development

#### Dataset preparation:

**Figure 3:**
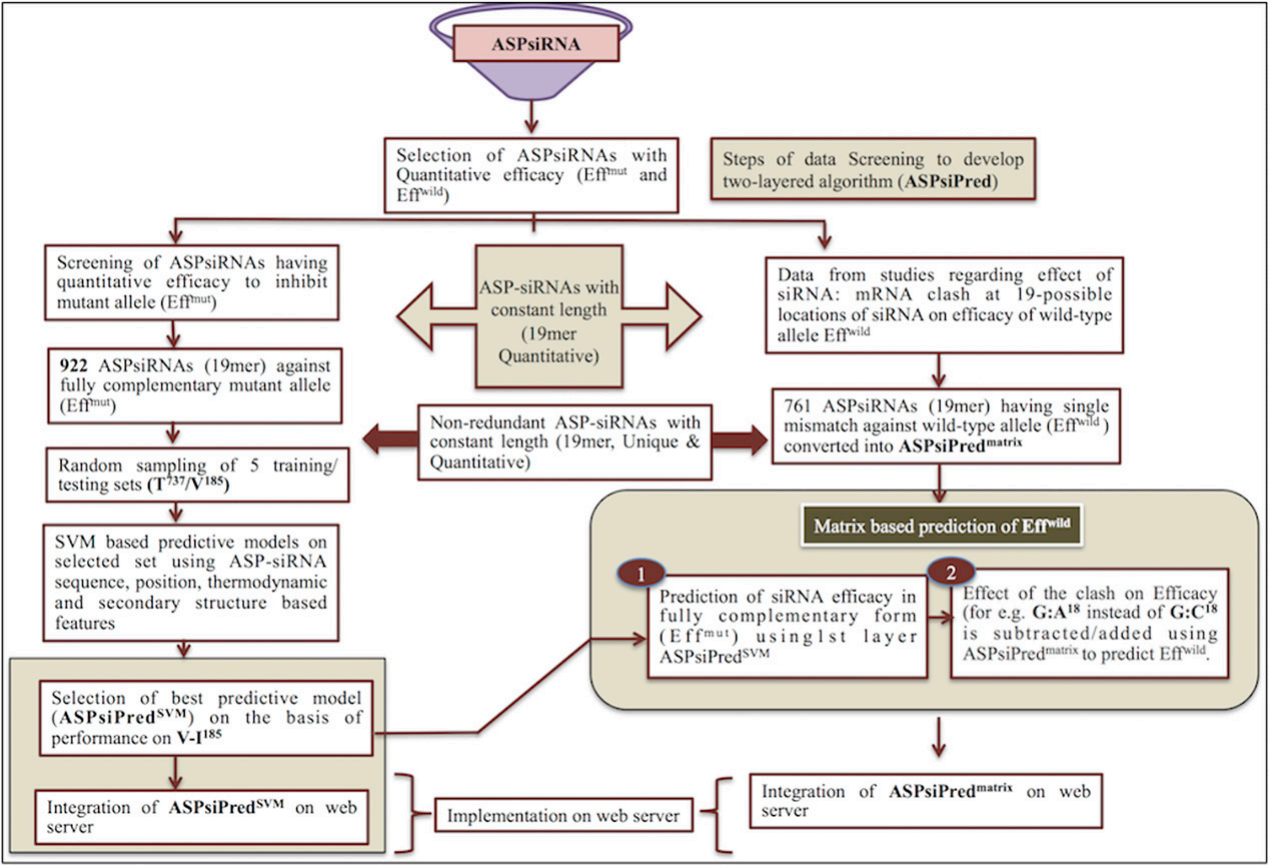
Computational workflow employed to extract ASP-siRNAs and developing the algorithm for the prediction of inhibitory efficacy: left arm describes the development of the SVM-based algorithm (ASPsiPred^SVM^) for prediction of efficacy for fully complementary mutant allele (Eff^mut^), while the right arm depicts the process of making ASPsiPred^matrix^ for the prediction of the efficacy for wild-type allele having one mismatch (Eff^wild^).

Since designing effective and discriminatory ASP-siRNAs is associated with two efficacy values, *i.e.*, one for a fully complementary target allele and a second for the nontarget allele, we have integrated a two-tiered algorithm in ASPsiPred (*ASPsiPred^SVM^* and *ASPsiPred^matrix^*) to predict Eff^mut^ and Eff^wild^, respectively (Figure 3).

In the first layer, *i.e.*, ASPsiPred^SVM^, we have screened ASPsiDb with 4543 ASP-siRNAs to get a unique and representative working dataset. After removing the 422 chemically modified (cm) ASP-siRNAs, we have processed the remaining 4121 sequences to extract 922 nonredundant 19mer siRNA sequences with quantitative efficacies (D922) (Supplemental Methods Section V and Table S2 in File S1). From D922, we have randomly extracted 185 sequences as independent/validation datasets (V185), while the remaining 737 sequences were used for the 10-fold cross-validation (10nCV) training/testing datasets (T737) (Tables S3 and S4 in File S1). This process was repeated five times to generate five training/testing and external validation sets.

#### Features used for model development:

Nucleotide composition and position-related features, thermodynamic stability and secondary structure based features were used in this study (see Supplemental Methods Section VII in File S1). We have selected these models/features and applied 10nCV on these sets. Once we obtained optimal results on selected hyper-parameters, we applied 10nCV on the full T922 dataset as a final classifier (Table S4 in File S1).

#### Algorithm development and validation:

The SVM*^light^* (http://svmlight.joachims.org) software package was used to train the different siRNA features and develop predictive models using 10nCV. In this study, we have used the radial basis function kernel for development of *ASPsiPred^SVM^*. We have evaluated the performance of our models using the Pearson correlation coefficient (PCC) (Supplemental Methods Section VIII and IX in File S1).

For the prediction of Eff^wild^, *i.e.*, the efficacy to inhibit target sequences with one mismatch, we have developed ASPsiPred^matrix^ (Tables S5–S8 in File S1) utilizing data from the following articles (Birmingham *et al.* 2006; Huang *et al.* 2009; Ohnishi *et al.* 2008; Schwarz *et al.* 2006) (Supplemental Methods Section X in File S1).

#### Implementation of ASPsiPred webserver:

ASPsiPred was developed on a SUN server using PERL, HTML, and CGI-PERL (Qureshi *et al.* 2013; Thakur *et al.* 2012a). Upon clicking ASPsiPred, a user is asked to enter the target and wild-type allele in FASTA format with the nucleotide mutation in lower case. For user convenience, we have provided a clickable example sequence. Our tool will generate ASP-siRNAs against mutation at all possible 19 locations followed by the prediction of Eff^mut^ and Eff^wild^ using ASPsiPred^SVM^ and ASPsiPred^matrix^.

**Figure 4:**
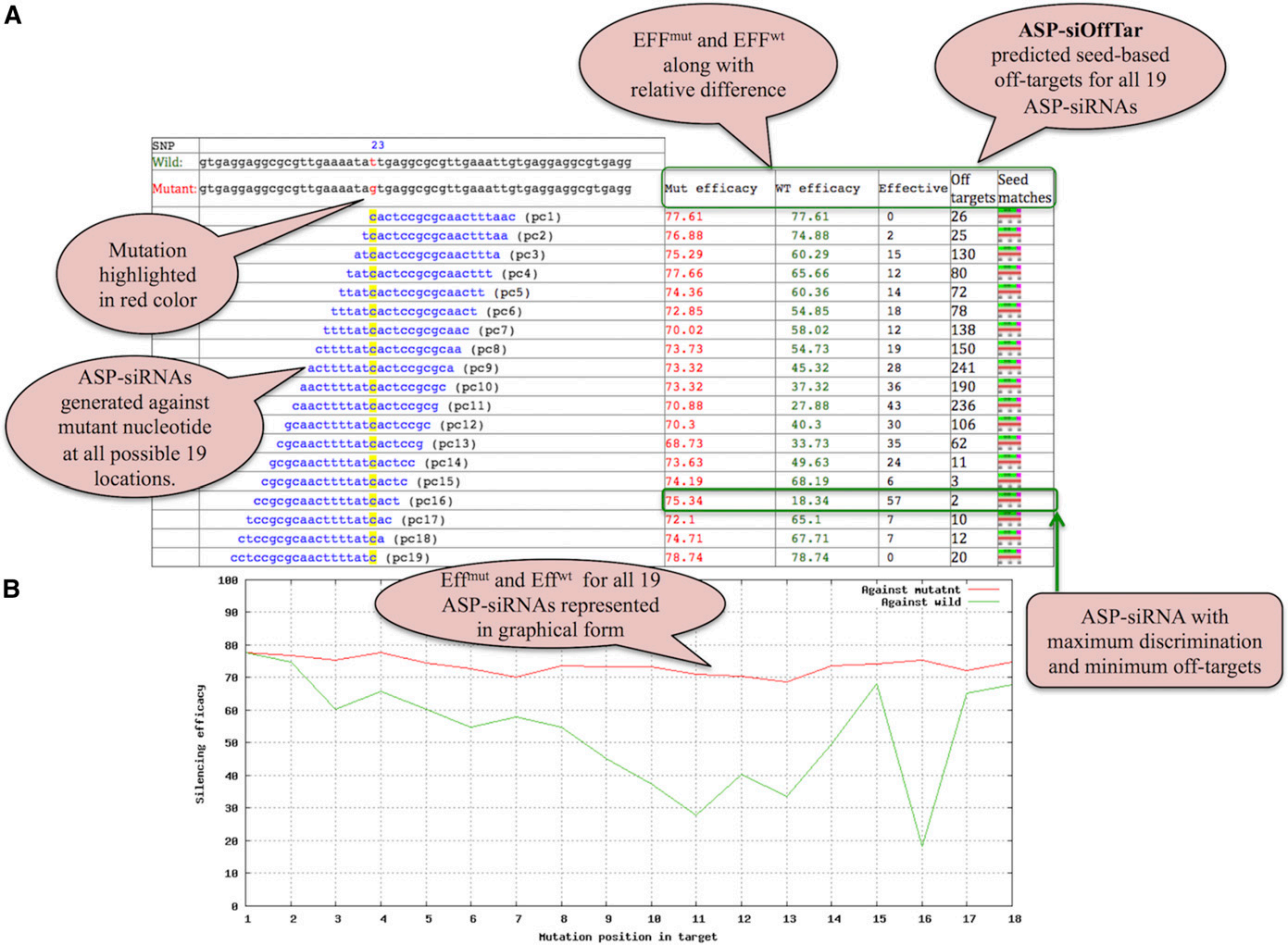
Description of ASPsiPred web server with result output. (A) Screenshot demonstrating ASP-siRNAs generated against a T > G mutation at all possible 19 positions along with Eff^mut^ and Eff^wild^ predicted from ASPsiPred^SVM^ and ASPsiPred^matrix^, respectively. Their relative difference between the two efficacies is also displayed along with the prediction of seed-based off-targets for all 19 ASP-siRNAs. (B) The output of the Eff^mut^ and Eff^wild^ of 19 ASP-siRNAs in graphical form.

We have integrated the ASP-siOffTar tool on the output page to provide seed-based off-targets for all predicted 19 ASP-siRNAs against user-provided mutation. This will give an idea about the potency as well as specificity of ASP-siRNA (Figure 4A). Thus, a user can select optimal allele-differentiating siRNAs with minimum off-target effects. The result is also displayed in a graphical format to analyze at which position ASP-siRNA displays relatively high discrimination for both alleles (Figure 4B).

### Analysis tools

#### ASP-siOffTar (seed based):

This provides a list of off-targets based on the alignment of hexamer (2–7) or heptamer (2–8) seed regions of ASP-siRNA or any siRNA on the human genome (build GRCh37). Since off targeting is majorly associated with the presence of perfectly complementary 3′-UTR matches with the seed region of the antisense strand of the siRNA (Birmingham *et al.* 2006), we have not allowed any mismatch in the alignment of seed regions on the human genome (Figure S6 in File S1).

#### ASP-siOffTar (full sequence based):

Full sequence based off-targets are also integrated as a separate tool on the web interface with a maximum of three allowed mismatches (Figure S7 in File S1).

#### ASP-siRNA-BLAST:

This matches a user-provided siRNA sequence against the ASPsiRNA database to find out whether similar siRNA/s are already reported.

#### ASP-siMAP:

Experimental biologists who seek to design an ASP-siRNA on their target gene can take advantage of the ASP-siMAP tool. It simply maps ASP-siRNAs reported in our archive to a user-specified target gene along with its start position.

### Data availability

All the data necessary for the results and conclusions in this paper are provided in the article or ASPsiRNA repository (http://crdd.osdd.net/servers/aspsirna/).

## Results

### ASPsiDb

#### Database statistics:

ASPsiDb is a manually curated and highly annotated depository of 4543 experimentally validated ASP-siRNA entries including 422 *chemically modified* (*cm*) ASP-siRNAs affecting 78 unique genes causing 51 various diseases out of which hemolytic uremic syndrome, HD, ALS, cancer, and PD were the top five diseases targeted (Figure S8a in File S1). Likewise, the *CD46* gene followed by *HTT*, *SOD1*, *DBI*, and *PPIB* were the top five genes (Figure S8b in File S1).

ASP-siRNAs were transfected using diverse transfection reagents; out of these lipofectamine 2000 was the most commonly used. Among the various methods reported to deliver ASP-siRNAs to the target locus, transfection (87.80%) was the major delivery method followed by shRNA expression vector (19.85%), lentiviral vector (1.66%), electroporation (1.38%), stereotaxic injection (0.76%), atelocollagen (0.57%) mediated delivery, and other methods (0.42%) (Figure S9 in File S1).

**Figure 5:**
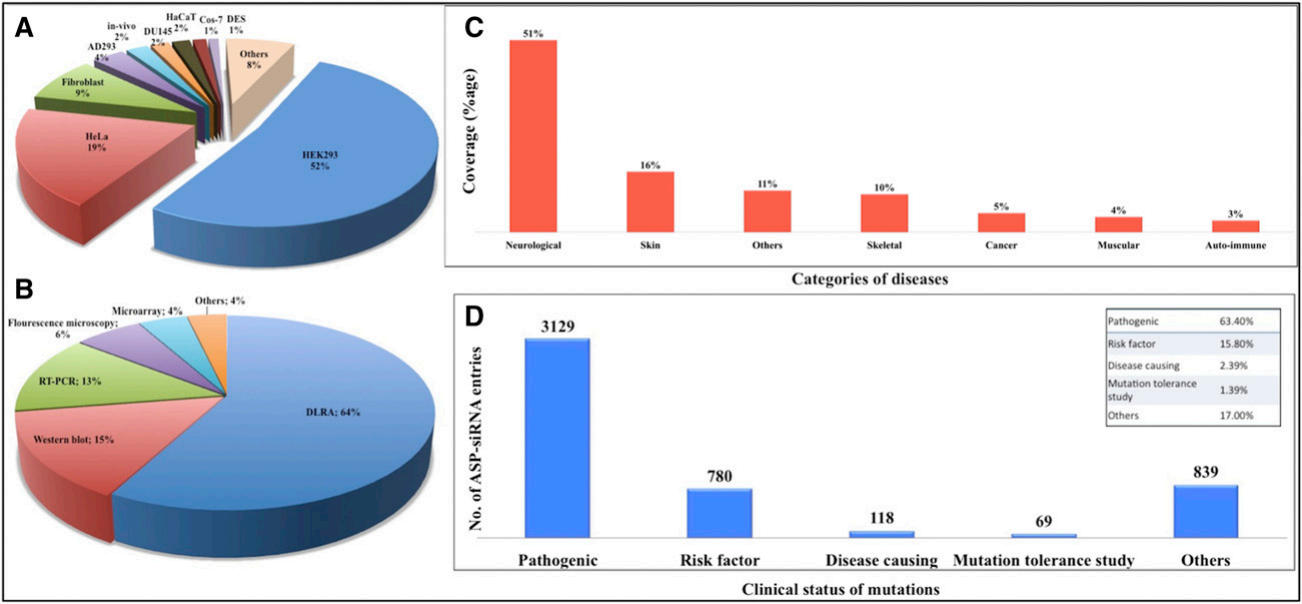
ASPsiRNA database statistics. (A and B) Pie charts exemplifying the distribution of cell lines and experimental methods used for validation of ASP-siRNAs. (C and D) Bar graphs describing the percentage coverage of different categories of genetic diseases and the statistical distribution of the clinical significance of diverse types of gene variants reported in the archive, respectively; described in ASPsiRNA.

The efficacy of various ASP-siRNAs was determined using 45 different cell-lines, among them HEK followed by HeLa, fibroblast, AD293, DU145, and HaCaT were most frequently used (Figure 5A). Animal models were also employed for *in vivo* studies including the transgenic mouse model, male Wistar rat, and *Caenorhabditis elegans*, out of which the mouse model was most common. In a particular study, human plantar calluses were also used to assess the potency of ASP-siRNA TD-101 targeting PC in a phase1b clinical trial (Leachman *et al.* 2010). Both RNA and protein level experimental methods were used for evaluating the efficacy; however DLRA (dual luciferase reporter assay) was reported in the majority of studies followed by western blot, RT-PCR, fluorescence microscopy, and microarray (Figure 5B).

Dominant genetic disorders are ideal candidates of ASGS due to its capability to target mutant alleles selectively. Our resource covers these disorders from seven different categories namely neurological disorders (ND) (51%), followed by skin (16%), skeletal (10%), cancer (5%), muscular disorders (4%), autoimmune diseases (3%), and others (11%) as depicted in Figure 5C.

For the design of effective and specific ASP-siRNAs, we have to select such an siRNA that causes least harm to the wild-type allele while keeping the mutant allele inhibition at the maximum level and displaying optimum allele discrimination (Davidson and Paulson 2004). Therefore, to analyze and find the discriminatory siRNAs, we have plotted the Eff^mut^ *vs.* the Eff^wild^ efficacies in the form of a scatter plot (Figure S10 in File S1). Statistical inspection reveals that the lower right section of the plot is quite dense as compared to the other quartiles. This section represents a high Eff^mut^ but low Eff^wild^. Thus, these sequences exhibit experimentally validated allelic discrimination most helpful for experimental biologists to target specific mutant alleles.

#### Statistical analysis of gene variants/mutations:

**Figure 6:**
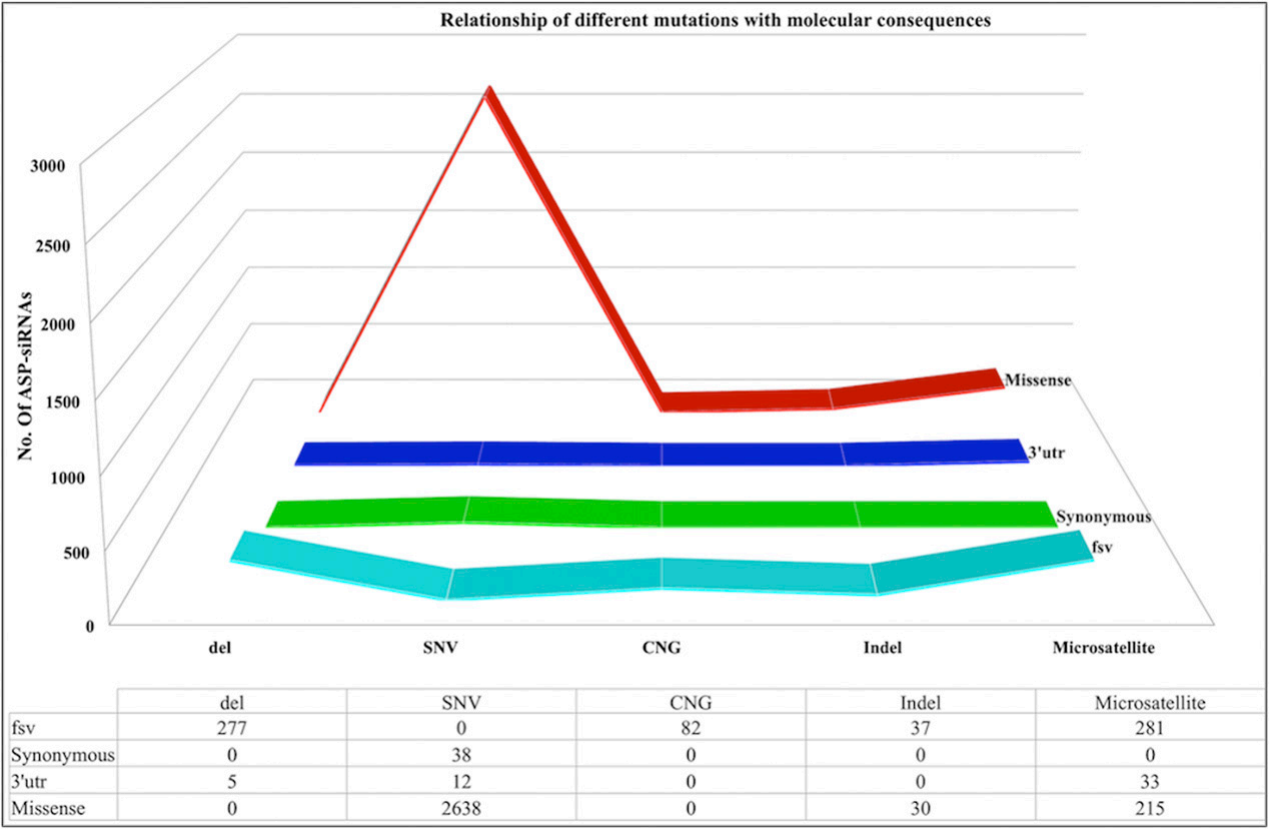
Different mutations and molecular consequences represented by 3D line-graph. fsv, frame shift variation; del, deletion; CNG, copy number gain; Indel, insertion-deletion.

We have analyzed the pathogenic status of various gene variants/mutations and found that ∼64% of ASP-siRNAs target pathogenic mutations (Figure 5D). We have also sketched all mutations and their associated molecular changes collected from ClinVar in the form of 3D-line graphs represented in Figure 6. It shows the statistical distribution of different sequence variations such as single-nucleotide variation (*snv*), microsatellite (expansion mutations), deletion (*del*), copy number gain (*CNG*), and insertion-deletion (*InDel*), which are associated with molecular consequences like missense mutation, frame shift variation (*fsv*), synonymous mutation, and 3′-UTR variant (variation in 3′ UTR region). Investigation of the graph indicates that: (i) in siRNAs targeting *snvs*, the molecular consequence is missense mutation in ∼98% of the cases; (ii) similarly, siRNAs targeting deletion variants cause *fsv* in ∼98% of cases; and (iii) siRNAs targeting microsatellite mutations mostly have a tendency to show *fsv* and missense mutations.

**Figure 7:**
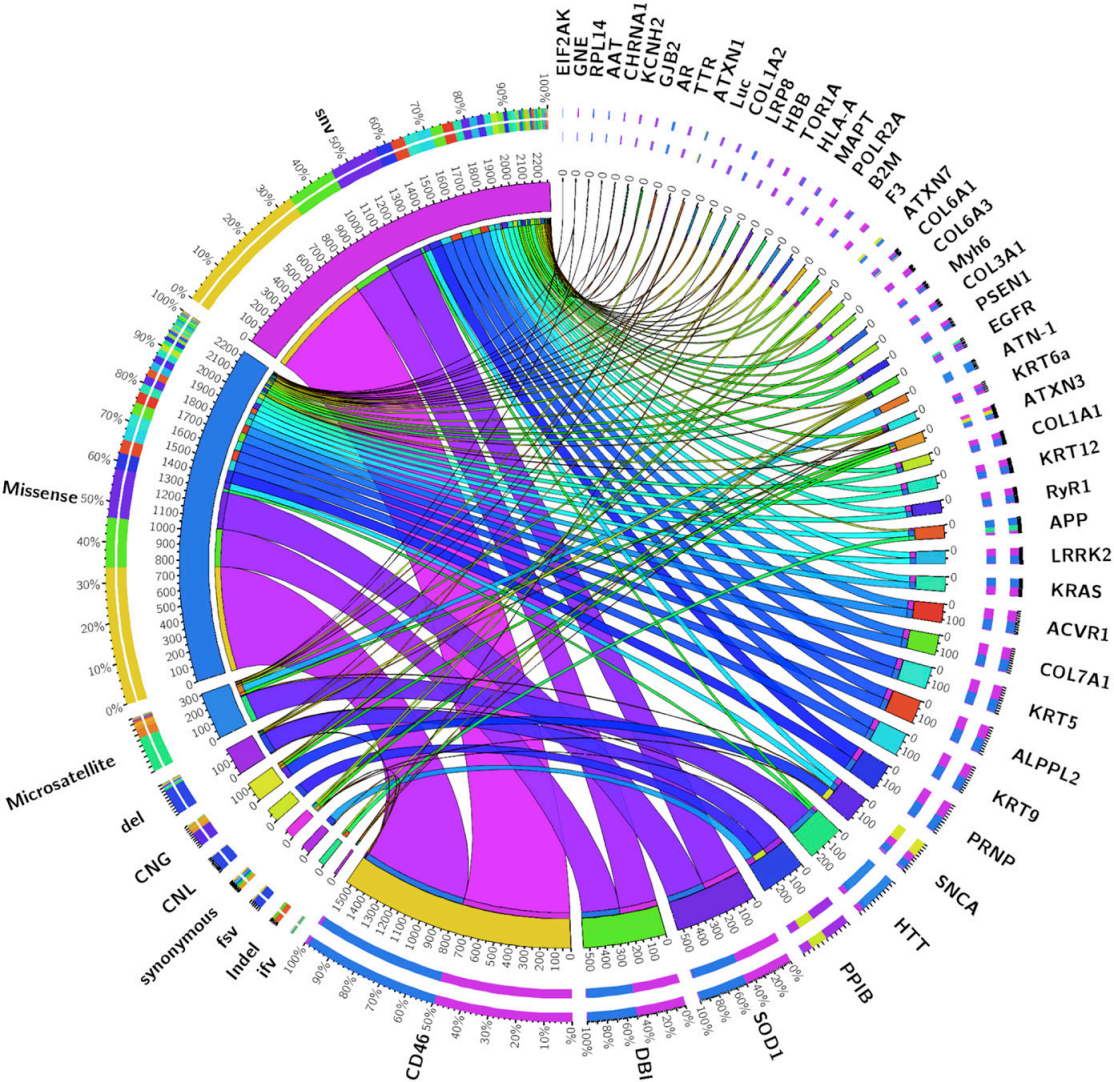
Mutational landscape of different genes described in ASPsiRNA epitomized by a circos plot: left and right hemi circle represents the mutation categories and gene names, respectively. The length of the main circular segments is proportional to the total number of ASP-siRNAs belonging to that segment, while the width of the ribbon connecting the gene with the mutation represents the proportion of ASP-siRNA sequences belonging to the particular mutation type. The two outer rings are contribution tracks, *i.e.*, stacked bar plots with a gradient of color signifying the proportion of entries from different genes.

A mutational landscape was summarized to investigate all gene variants/mutations examined by ASP-siRNAs with the help of circos plot (Krzywinski *et al.* 2009). It shows that ASP-siRNAs mostly target genes that had single-nucleotide substitutions (SNPs) and missense mutations (Figure 7). This observation is in accordance with the Human Genome Database (HGDB), which states that out of 73,411 reported mutations responsible for causing genetic diseases, >60% are caused by SNPs (Seyhan 2011).

### ASPsiPred performance evaluation

#### ASPsiPred^SVM^: performance during 10nCV:

Selected sequence features (mdtt+binary) (see Supplemental Methods Section VII in File S1) were used to perform 10nCV on five random training/testing sets (T737). Their performance was measured on an independent validation dataset (V185) (Table S3 in File S1). After confirming that all five sets performed approximately similarly, we have selected Random Set-2 to build final classifier without any bias (random set-2).

During 10nCV on the selected set, predictive models based upon sequence composition based features like mono-, di-, tri-, tetra-, and penta-nucleotide compositions achieved a maximum correlation of 0.53, 0.68, 0.70, 0.69, and 0.68, respectively. Position-based features like the binary pattern of nucleotides attained a PCC of 0.55. We have also developed *hybrid models* using >1 nucleotide features as input, *e.g.*, hybrid of mono- (m) and dinucleotide (d) composition (*md*). We achieved correlations of 0.67, 0.70, 0.71, 0.71, 0.71, and 0.71 in the md, mdt, mdtt, mdttp, mdtt + binary, and mdttp + binary hybrid models, respectively (see Table 1). Accordingly, performance of thermodynamic and secondary structure based features achieved a PCC of 0.41 and 0.24, respectively; however, their hybrid with our best model did not lead to an improvement in correlations (Table 1, model 12+13, 12+14, and 12+13+14). The sequence features, which performed best on set-2, *i.e.*, ASPsiPred^SVM^ (mdtt + binary), were applied to the total dataset (D922) as a final classifier on the webserver termed as ASPsiPred^SVM#^ (Table S4 in File S1).

**Table 1:** Performance of different predictive models on the training/testing dataset of 737 sequences (T737) during 10-fold cross-validation. Evaluation of the models on an independent validation dataset (V185)

			PCC on Training/Testing Sets (T737) and Independent Validation Sets (V185) Using 10nCV	
Predictive Model No.	siRNA Feature Name	No. of Features	T737	V185
1	Mononucleotide composition	4	0.53	0.54
2	Dinucleotide composition	16	0.68	0.64
3	Trinucleotide composition	64	0.70	0.66
4	Tetranucleotide composition	256	0.69	0.65
5	Pentanucleotide composition	1024	0.68	0.63
6	Binary	76	0.55	0.56
7	1+2	20	0.67	0.63
8	1+2+3	84	0.70	0.63
9	1+2+3+4	340	0.71	0.65
10	1+2+3+4+5	1364	0.71	0.65
11	1+2+3+4+6 (ASPsiPred^SVM^)	416	0.71	0.65
12	1+2+3+4+5+6	1440	0.71	0.65
13	Thermodynamic feature	21	0.41	0.30
14	Secondary structure	19	0.24	0.07
15	13+14	40	0.35	0.23
16	12+13	437	0.71	0.65
17	12+14	435	0.71	0.65
18	12+13+14	456	0.71	0.65
19	ASPsiPred^matrix^	Matrix based	Developed on rules-based studies	0.63

PCC, Pearson correlation coefficient; 10nCV, 10-fold cross-validation; T737, training/testing dataset for 10-fold cross-validation; V185, independent validation dataset. PCC is between actual and observed Eff^mut^. Training/testing dataset is used to train different predictive models, while independent validation dataset was not used anywhere during training/testing of algorithm.

##### Performance on independent validation dataset (V185):

The performance of the predictive models was assessed on V185. Our best model achieved a maximum (PCC) of 0.71 during 10nCV on the training dataset (T737) termed as *ASPsiPred^SVM^*. On V185, a comparable PCC of 0.65 was obtained (Table 1). Scatter plots depicting the correlation between the actual and predicted efficacy during 10nCV and independent validation are shown as Figures S11 and S12 in File S1.

##### Performance during leave one target out cross-validation (LOTOCV):

Since D922 contains sequences having single-nucleotide sliding difference (see more in Supplemental Methods Section VI in File S1), a simple 10nCV on random training/testing dataset in which some sequences are in the training dataset while others are in the test set can inflate the performance of classifier. Therefore, to deal with overlapping sequences and to check the predictive contribution of each target gene in the D922, we have used the LOTOCV method.

In this method, we have assigned ASP-siRNAs targeting a particular gene in the validation dataset, while sequences from other genes were assigned to the training set. In total, 22 different sets have been made including one heterogeneous set titled “Others” which includes genes for which fewer ASP-siRNAs (<10) were reported (Table 4). Overall performance during 10nCV ranged from PCC values of a minimum of 0.53 to a maximum of 0.74 with an average PCC of 0.66. Performance on validation sets ranged from a PCC value of 0.20 to 0.88 with an average PCC of 0.40.

##### Comparison of ASPsiPred^SVM^ with other webservers:

While comparing the performance of any two algorithms, one should use the same dataset for training and testing (Ahmed and Raghava 2011). In the literature, second-generation siRNA efficacy prediction tools were developed using the Huesken dataset and exhibit a very good PCC in the range of 0.56–0.85 (Train# column of Table 2). On the other hand, ASPsiPred^SVM^ is developed on an updated ASP-siRNA dataset. Therefore, finding no similarity in the datasets employed to develop these tools, we have done comparative evaluation in three ways, *i.e.*, by assessing the performance of (i) our algorithm with previously developed methods, (ii) *cross-replacement* of datasets, and (iii) our algorithm on an independent benchmarking dataset designated as “V419” (Ichihara *et al.* 2007).

**Table 2:** Performance of second-generation siRNA efficacy prediction algorithms on T737, V185, and V419

					Pearson Correlation Coefficient (PCC)				
S. No.	Reference	Technique	siRNA Dataset	ASP-siRNA Dataset	Train#	Val#	T737	V185	V419*
1	Huesken *et al.* (2005)	ANN	Huesken^2431^	✗	0.67	0.66	Webserver not working		0.54
2	Vert *et al.* (2006)	LR	Huesken^2431^	✗	0.67	0.57	Webserver not working		0.55
3	Jiang *et al.* (2007)	RFR	3589	✗	0.85	0.59	Webserver not working		NA
4	Ichihara *et al.* (2007)	LR	Huesken^2431^	✗	0.72	NA	***0.18***	***0.14***	0.56
5	Ahmed and Raghava (2011)	SVM	Huesken^2431^	✗	0.65	0.65	***0.27***	***0.25***	0.55
6	siRNApred Kumar *et al.*, (2009)	SVM	Huesken^2431^	✗	0.56	0.47	***0.27***	***0.09***	0.23

Second-generation siRNA efficacy algorithms were developed on the Huesken dataset. S.No., Serial number; RFR, random forest regression; ANN, artificial neural network; LR, linear regression; Train^#^ and Val# is the performance during n-fold cross-validation and independent validation of a particular algorithm. T737 and V185 column reflects the performance of algorithms on training/testing and independent validation sets of ASPsiPred^SVM^ (in bold italics), while extreme right column indicates performance of algorithms on benchmarking dataset V419.

Our best model has achieved a maximum PCC of 0.71 on 10nCV and 0.65 on independent validation; which is comparable to previously developed siRNA efficacy prediction methods (Table 1). In the cross-replacement strategy, we have assessed the performance of available algorithms on our dataset (Table 2) and ASPsiPred^SVM^ on theirs (Table 3). We found algorithms developed on Huesken^2431^ achieved PCCs in the range of 0.18 to 0.27 and 0.09 to 0.25 on our T737 and V185 datasets, respectively (see Table 2). On the other hand, ASPsiPred^SVM^ has achieved PCCs of 0.23 and 0.26 on Huesken^2431^ (T^2182^/V^249^) (Table 3).

**Table 3:** Performance of ASPsiPred^SVM^ on Huesken^2431^ and V419

S. No.	Reference	Technique	siRNA dataset	ASP-siRNA	T737	V185	T2182	V249	V419
1	ASPsiPred^SVM^	SVM	ASP-siRNA (D922)	✓	0.71	0.65	0.23	0.26	0.22

S.No., Serial number. The Huesken^2431^ dataset is divided into T2182 and V249 as training/testing and independent validation set. T737 and V185 column reflects the performance of ASPsiPred^SVM^ on training/testing and independent validation sets; while V419 indicates performance on benchmarking dataset.

Further, we have checked the performance of our algorithm on an independent benchmarking dataset, V419 (Ichihara *et al.* 2007). This dataset has also been utilized in previous tools to assess their performance. While Huesken-based methods have achieved correlation of 0.23 to 0.56 on V419 (extreme right column in Table 2), we attained a PCC of 0.22 (Table 3).

#### ASPsiPred^matrix^: performance evaluation of ASPsiPred^matrix^ on validation datasets:

The second tier of our algorithm is the mismatch information matrix generated from the rule-based studies. It had achieved a PCC of 0.63 on V185 (Table S8 in File S1).

#### Comparison of ASPsiPred^Matrix^ with other webservers:

Currently, there is no webserver to predict Eff^wild^, although one method desiRm exists that describes the improvement in the efficacy of an siRNA after introducing mismatches in it. On the other hand, our method has the same ASP-siRNA but assessed against mismatches with the wild-type allele. Therefore, we have compared the performance of both methods using four experimental studies in which 19mer ASP-siRNAs complementary to a sliding window across a mutation were assessed. Performance of desiRm was not satisfactory on single-nucleotide sliding trails, while the matrix-based method attained a collective PCC in the range of 0.35–0.52 (Table S8 in File S1).

## Discussion

Post-ENCODE (Lussier *et al.* 2013; Venter *et al.* 2001), a plethora of information has been released about genome sequence, structure and multifaceted ways of its regulation. This information has provided new opportunities to understand complex genetic disorders at the molecular level. Thus, it will be useful for tailoring the conventional gene therapy into a custom-made one (Lander 2011). In this context, RNA targeting approaches up to the precision of single-nucleotide discrimination are emerging as a potential and therapeutic alternative to traditionally undruggable targets (Keiser *et al.* 2016).

ASGS is a progressive technique for tailored treatment of dominantly inherited disorders. An ASP-siRNA is designed to target an allele of interest/mutant allele at any location where it differs from its wild-type counterpart (Lombardi *et al.* 2009). Despite its immense medical importance, a dedicated informatics resource in this field was lacking, which encouraged us to develop resources on ASP-siRNAs implicated in various genetic diseases. While existing archives hold information about siRNAs targeted against one gene with a single inhibitory efficacy (Table S1 in File S1), *ASPsiDb* harbors ASP-siRNAs targeted against the mutant and wild-type alleles of a gene and hence associated with two inhibitory efficacies (Eff^mut^/Eff^wild^).

It was after the breakthrough discovery that RISC-mediated cleavage occurs at the phosphodiester bond of the 10th nucleotide position on the guide strand (Elbashir *et al.* 2001b; Haley and Zamore 2004) that researchers around the world started utilizing its role in achieving ASGS by placing the nucleotide complementary to the mutation at the 10th or central positions of siRNAs to make it less accessible to the normal allele. This scrutiny was employed in achieving ASGS by directly targeting disease-causing mutations (Jiang *et al.* 2013; Lyu *et al.* 2016) or indirectly targeting disease-associated SNPs in linkage disequilibrium (Drouet *et al.* 2014; Yu *et al.* 2012). Moreover, mutation-specific suppression has also been accomplished for mutant alleles exhibiting deletions by placing mutation-specific nucleotides at the central positions (Gonzalez-Alegre *et al.* 2003). Although there were several reports studying the effect of placing nucleotides complementary at the mutation on the efficacy of the mutant allele (Eff^mut^), but an algorithm employing these studies was lacking.

Correspondingly, there were some rule-based studies reporting the effect of *siRNA*: *mRNA residue clash* on efficacy at all 19 locations of the siRNA guide strand (Birmingham *et al.* 2006; Huang *et al.* 2009; Ohnishi *et al.* 2008; Schwarz *et al.* 2006). It is also testified that *siRNA*: *mRNA residue clash* of purine: purine (*pur*:*pur*) type is less tolerable than pyrimidine: pyrimidine (*pyr*:*pyr*) clash. For example, siRNA “siC7/8” having G: G clash with the wild-type allele suppresses the mutant allele three fold more than its counterpart (Miller *et al.* 2003). In some cases, when siRNA: mRNA have a *pyr*:*pyr* or *pyr*:*pur* clash, an additional mismatch is introduced in the siRNA to make it more discriminative (Miller *et al.* 2004). Despite these rule-based studies, there is no algorithm employing these findings for prediction of Eff^mut^ and Eff^wild^. We have developed ASPsiPred, the first web server in this field incorporating a two-tiered algorithm (ASPsiPred^SVM^ and ASPsiPred^matrix^) for predicting efficacies Eff^mut^ and Eff^wild^.

In the literature, initially many mammalian siRNA efficacy prediction algorithms were developed using heterogeneous siRNA datasets and achieved a good PCC of 0.46–0.56 (Holen 2006; Saetrom 2004; Shabalina *et al.* 2006). Thereafter, algorithms to predict siRNA efficacies were reported using the Huesken dataset (Huesken *et al.* 2005) and exhibited very good PCC values in the range of 0.56–0.85. Likewise, ASPsiPred^SVM^ has achieved a correlation of 0.71 on 10nCV and 0.65 on an independent validation set (Table 1). The ASP-siRNA dataset (D922) has not been employed anywhere in the present mammalian siRNA efficacy algorithms. Moreover, our algorithm has not utilized currently available siRNA datasets other than D922. Further, it has been reported that siRNA algorithms perform less well on datasets in which they have not been trained (Qureshi *et al.* 2013). Correspondingly, the performance of other available algorithms on our dataset (Table 2) and ASPsiPred^SVM^ on their datasets was lower (Table 3).

ASPsiPred^SVM^ performed better on the ASP-siRNA datasets including T737 and V185 sets (Table 3). However, it achieved a PCC of 0.23 and 0.26 on the Huesken^2431^ dataset (T^2182^/V^249^). This may be because it has only been trained on an allele-specific dataset and suggests the need of an ASP-siRNA efficacy prediction algorithm. Thus, ASPsiPred^SVM^ will be helpful for researchers in designing and predicting Eff^mut^ for consecutive single-nucleotide sliding siRNAs for a given gene that is not necessarily linked to disease. For this purpose, we have provided our best predictive model as a general siRNA efficacy predictor under the separate ASPsiPred^SVM^ section on the web server.

As the D922 dataset covers sequences with single-nucleotide sliding differences, there is overlap among them. Therefore, the simple 10nCV in which overlapping sequences are randomly assigned to training and test sets could inflate the performance of the algorithm. Thus, to further address this issue, we have used the LOTOCV method in which ASP-siRNAs from each target gene are iteratively excluded and the classifier is trained on sequences from the remaining genes followed by testing on the sequences from the excluded gene (Table 4). Out of the 21 genes, predictive performance of 14 genes was satisfactory despite the fact that data from that gene were not present in the training set. Therefore, results from the above strategy show that ASPsiPred^SVM^ can act as a general ASP-siRNA efficacy prediction algorithm for other genes (Table 4). However, predictive performance of some of the genes was less than satisfactory. This may be due to the difference in the pattern of the target gene mutation, which might be improved in the future based on the availability of more data.

**Table 4:** Performances of the SVM models during 10-fold cross-validation using LOTOCV method

		No. of ASP-siRNAs		Pearson Correlation Coefficient (PCC) During 10nCV and IV	
S. No.	Gene Name	Training Dataset	Validation Dataset	10nCV	IV
1	APP	907	15	0.71	0.88
2	AR	912	10	0.71	0.19
3	COL1A1	912	10	0.71	0.49
4	COL3A1	903	19	0.71	0.34
5	COL6A3	911	11	0.70	0.24
6	COL7A1	903	19	0.71	0.55
7	HTT	883	39	0.56	0.28
8	KRAS	844	78	0.68	0.31
9	KRT12	884	38	0.71	0.48
10	KRT5	884	38	0.71	0.24
11	KRT6a	903	19	0.70	0.31
12	KRT9	830	92	0.63	0.26
13	LRRK2	901	21	0.71	0.26
14	Others	844	78	0.74	0.20
15	P. Luciferase	865	57	0.71	0.23
16	PPIB	695	227	0.53	0.61
17	PRNP	904	18	0.71	0.79
18	PSEN1	903	19	0.43	0.30
19	SNCA	906	16	0.71	0.50
20	SOD1	881	41	0.53	0.34
21	TGFBI	903	19	0.55	0.64
22	TP63	884	38	0.58	0.33

ASP-siRNAs targeting a particular gene are assigned to the validation dataset, while sequences from other genes were assigned to the training set. Validation of the models was done using respective gene in the independent validation set. Standard HGNC gene symbols have been used. PCC is between the actual and observed Eff^mut^. The training dataset is used to train different predictive models, while independent validation datasets were not used in any training algorithms. S.No., Serial number; 10nCV, ten-fold cross-validation; IV, independent validation.

Additionally, there is no web server to predict the efficacy of ASP-siRNAs with a wild-type allele having a single mismatch (Eff^wild^). Though desiRm also deals with mismatches and efficacy, it aims to improve the efficacy of an siRNA by introducing mismatches in the same target sequence. On the other hand, ASPsiPred^matrix^ is intended to predict the efficacy of ASP-siRNA targeting a wild-type allele (Eff^wild^) with one mismatch. desiRm is associated with one efficacy value at a time, while ASPsiPred predicts two efficacies (Eff^mut^/Eff^wild^) simultaneously from two methods. In the former, a mismatch is introduced in the siRNA for the same target sequence to improve efficacy, while in the latter case, a mismatch is present between wild-type allele and ASP-siRNA. desiRm was developed on the Huesken dataset and ASPsiPred is developed using ASPs-RNAs, which is a novel siRNA dataset in the literature. We have also compared the performance of both methods on four experimental studies of multiple 19mer siRNAs offset along a target and found that ASPsiPred^matrix^ performs better in predicting single-nucleotide sliding 19mer trails (Table S9 in File S1).

It is well established that off-target effects are a major issue during siRNA-based gene silencing and seed regions are a key determinant for these effects (Birmingham *et al.* 2006; Jackson *et al.* 2003; Kamola *et al.* 2015). Therefore, to deal with off-targets, we have also integrated the *ASP-siOffTar* tool to deliver a list of off-target hits based on the alignment of the seed regions of ASP-siRNA or any siRNA to the human genome. To extend the off-targets repertoire of particular siRNAs, a full sequence based off-target tool is also integrated on the web interface with a maximum of three allowed mismatches. Furthermore, many chemical modifications (cm) on siRNAs have been used to reduce off-target effects and increase the half-life of siRNAs by making it nuclease resistant (Dar *et al.* 2016b). We have also compiled a list of 422 cm ASP-siRNAs and provided it on our web server.

Although ASP-RNAi is a powerful tool, various factors must be taken into account before it enters clinic, such as binding of siRNAs to unintended off-targets via partial sequence complementarity (Kamola *et al.* 2015), stability, and half-life (Dar *et al.* 2016b). Successful siRNA delivery is also an important contributing factor, which depends upon choice of transfection reagent and the intrinsic susceptibility of the target cell type (Nabzdyk *et al.* 2011).

Thus, the ASPsiRNA resource would be immensely helpful for *in silico* design and predicting efficacy of ASP-siRNAs for various maladies, *e.g.*, in cancer-associated SNPs (Iyer *et al.* 2016; Mook *et al.* 2009), for treatment of genetic diseases, *e.g.*, from currently incurable autosomal dominant (Miller *et al.* 2004) to severe sex-linked disorders (Caplen *et al.* 2002), in combating viral drug resistance (Teng *et al.* 2011), and many more. It will also be beneficial for researchers who wish to study the function of alleles.

Currently, our method is limited to the prediction of Eff^wild^ with a single mismatch due to limited data on multiple mismatches. It also has limited performance on unseen or novel genes owing to a limited number of target genes in the dataset. In the future, there would be a need to develop an algorithm for >1 mismatch, which can improve allelic discrimination. Nevertheless, the upcoming use of ASP selectivity will not only be useful to suppress disease-associated SNPs, but can also be applied as a research tool where you can silence one splice variant from other (Trochet *et al.* 2015).

### Conclusion and future implications

Understanding distinctive aspects of ASGS by ASP-siRNAs may be exploited in the treatment of currently incurable dominant genetic disorders. In this ASPsiRNA resource, *ASPsiDb* provides a highly annotated dataset of ASP-siRNAs and their associated targets. It also provides a two-layered algorithm to design effective and discriminatory siRNAs against heterozygous SNPs (ASPsiPred^SVM^) and wild-type alleles (ASPsiPred^matrix^) coupled with useful tools like *ASP-siOffTar* for off-target analysis. We hope ASPsiPred will be immensely helpful to target not only disease-causing mutations, but also to study the biological function of alleles that are not necessarily linked to disease.

## Supplementary Material

Supplemental material is available online at www.g3journal.org/lookup/suppl/doi:10.1534/g3.117.044024/-/DC1.
