# Supplementary material for: ASPsiRNA: A Resource of ASP-siRNAs Having Therapeutic Potential for Human Genetic Disorders and Algorithm for Prediction of Their Inhibitory Efficacy
Source: G3 (Bethesda). 2017 Jul 6;7(9):2931–43. doi: 10.1534/g3.117.044024 (PMC5592921; doi:10.1534/g3.117.044024)
Supplement: Supplementary file 1 [file 2931FileS1.doc]

**ASPsiRNA: A resource of ASP-siRNAs having therapeutic potential for human genetic disorders and algorithm for prediction of their inhibitory efficacy**

Isha Monga, Abid Qureshi, Nishant Thakur, Amit Kumar Gupta and Manoj Kumar*

Bioinformatics Centre, Institute of Microbial Technology, Council of Scientific and Industrial Research, Sector 39-A, Chandigarh-160036, India

* To whom correspondence should be addressed: manojk@imtech.res.in

**Supporting information**

1. **Supplementary methods for database section**
2. ASP-siRNA data extraction
3. Clinical information regarding various mutations
4. ASPsiDb web interface: Searching and browsing
5. Details of individual ASP-siRNA ID
6. **Supplementary methods for Prediction algorithm section**
7. Dataset preparation: ASPsiPred^SVM^
8. **Overlap in D922 dataset:**
9. Features used for model development
10. Algorithm development
11. Validation
12. Dataset preparation: ASPsiPred^matrix^

**List of supplementary Tables**

**Table S1.** List of computational resources for experimentally validated siRNAs

**Table S2.** Steps involved in the extraction of 922 ASP-siRNAs dataset for ASPsiPred^SVM^

**Table S3.** Performance of random independent validation sets (V185) using SVM models developed on their respective training/testing sets (T737) employing 10-fold cross-validation (10nCV)

**Table S4.** Results of 10-fold cross-validation (10nCV) on complete dataset T922

**Table S5.** Mismatch discrimination profile made from rule-based studies for Guanine (G) at 19 possible locations of siRNA guide strand. This profile for Guanine was utilized in developing final matrix i.e. ASPsiPred^matrix^

**Table S6.** Mismatch discrimination profile constructed from different studies for Adenine (A) at 19 possible locations of siRNA guide strand

**Table S7.** Mismatch discrimination profile made from rule-based studies for Uracil (U) at 19 possible locations of siRNA guide strand

**Table S8.** Mismatch discrimination profile built from rule-based studies for Cytosine (C) at 19 possible locations of siRNA guide strand

**Table S9.** Overall comparison of ASPsiPred^matrix^ and desiRm using four different experimental studies having nucleotide complementary to mutation on across 19mer ASP-siRNA

**[Note:** Performance is measured by PCC between predicted and actual inhibitory efficacy of ASP-siRNA for wild-type allele with one mismatch (Eff^wild^)]

**List of supplementary Figures**

**Figure S1.** An example screenshot representing the search result for ASP-siRNAs with keyword “amyloid lateral sclerosis”

**Figure S2.** Screenshot demonstrating the first part of individual ASP-siRNAs record (this section harbors information on ASPsiRNA designed against mutant allele, location of mismatch on siRNA guide strand, gene and mutation location)

**Figure S3.** Screenshot demonstrating the second section of individual ASP-siRNAs entry, which entails data on various aspects of disease like pathogenicity, mutation in HGNC format and links to clinical resources

**Figure S4.** Figure representing the third section of individual record (this part is offering experimental data on Eff^mut^ and Eff^wild^ by ASP-siRNA and siRNA targeting wild-type allele)

**Figure S5.** Figure demonstrating the fourth part of information obtained for individual ASP-siRNA entry

**Figure S6.** Screenshot representing the search result for seed based ASP-siOffTar tool

(Note: as shown in screenshot, Ensembl ID of aligned target sequence is provided along with start and end position. No mismatch is allowed in seed-based off-targets alignment on human genome)

**Figure S7.** Screenshot representing the search result for full sequence based ASP-siOffTar tool (Note: as shown in screenshot, Ensembl ID of aligned target sequence is provided along with start and end position. A maximum of three mismatches are allowed in off-targets based on alignment of full siRNA sequence on human genome)

**Figure S8**. Bar graph depicting the statistical distribution of ASP-siRNAs for numerous human genetic diseases (a) and genes (b) reported in ASPsiDb (x-axis denotes human genes while y-axis signifies number of ASP-siRNAs)

**Figure S9.** Bar graph representing the distribution of different delivery methods reported in ASPsiDb (x-axis denotes various delivery methods while y-axis signifies number of ASP-siRNAs)

**Figure S10.** A scatter plot displaying percentage efficacy distribution of ASP-siRNAs for mutant (Eff^mut^) versus wild-type allele (Eff^wild^) of target gene (x-axis represents efficacy for mutant allele, while y-axis signifies efficacy for wild-type allele)

**Figure S11.** Smooth scatter plot in R depicting correlation between the actual and predicted percentage efficacy of T737 during 10nCV using SVM

**[Note-** T737: train/test set; 10nCV: ten-fold cross-validation**]**

**Figure S12.** Smooth scatter plot in R illustrating correlation between the actual and predicted percentage efficacy of V185 during 10nCV using SVM

**[Note:** V185: validation set; 10nCV: ten-fold cross-validation**]**

**Figure S13.** Smooth scatter plot in R representing correlation between the actual and predicted percentage efficacy of D922 during 10nCV using SVM

[Note: D922- Total ASP-siRNA dataset; 10nCV-ten-fold cross-validation]

**Figure S14.** Histogram with normal distribution function curve: Histogram illustrating percent efficacy range (x-axis) and frequency of total ASP-siRNA dataset (D922) (z-axis). Normal density distribution curve depicting bell shaped curve with slightly right skew between percent efficacy (x-axis) and normal density for each efficacy data point using normal density distribution function (y-axis)

**Figure S15.** Normality testing/outlier analysis by Z-value: scatter plot representing Z-value versus expected value (red line) and z-value versus originals (blue line)

[Note: Expected value is calculated from CDF (cumulative distribution factor), mean and std. deviation]

1. **Supplementary methods for database section**
2. **ASP-siRNA data extraction**

Advanced search retrieved 1267 articles till May 2017. After omitting population studies of different human genetic diseases, articles related to RNAi experiments on plants, allele-specific RNA-seq, reviews and general methodology papers; we shortlisted approximately 610 articles. Finally, data was extracted from 89 PMID’s and 6 patents. Information extracted from articles were ASP-siRNA sequence, its target sequence along with details on mutation position; efficacies to silence mutant and wild-type allele (Eff^mut^ and Eff^wild^) respectively. Further, general experimental details including experimental technique used to access efficacy of siRNA; cell-line; transfection reagent and its duration were also obtained. These Studies have utilized luciferase assay system for the assessment of ASP-siRNA specificity by engineering mutant/wild-type sequences into 3’ UTR of *Renilla/Firefly* luciferase vector (Liao et al., 2011; Lombardi et al., 2009; Muller et al., 2012; Ohnishi et al., 2006). Their relative expression is accessed in various cell lines using simple/dual luciferase reporter assay system. Though, dual luciferase is frequently exploited experimental technique, some studies have also utilized western blot analysis (Miller et al., 2004) and RT-PCR (Loy et al., 2012) to check the protein and RNA expression of target allele.

1. **Clinical information regarding various mutations**

Following query was used ‘*disease’* ‘AND’ *mutation* ‘OR’ *gene name* ‘OR’ ‘*variant*’ in ClinVar to extract clinical data pertaining to mutation given in the article. Different aspects of sequence variation were covered *viz* mutation category (for e.g. *single nucleotide variation* (*snv*)); its position in the particular cDNA, gene and protein; molecular consequence i.e. effect of the variant at molecular level (e.g. missense variant), clinical significance of the mutation (e.g. pathogenic/likely pathogenic) from ClinVar, refSNP, dbVar, COSMIC, DECIPHER OMIM etc.

1. **ASPsiDb web interface: Searching and browsing**

Search output provides a list of ASP-siRNAs matching with the input query; each ASP-siRNA is paired with its mutant allele displaying the percentage efficacies to silence mutant and wild-type allele. Additionally it presents important features of ASP-siRNAs e.g. target gene, disease, target mutation, Reference SNP (RefSNP), literature reference and hyperlink to the output of analysis tools (off-targets seed based and full sequence). Each column of the search output is provided with sorting and filtering functionality (**Fig. S1**).

1. **Details of individual ASP-siRNA ID**

***1) Information on ASP-siRNA***: includes ASP-siRNA ID and name. **2) *Mismatch information*:** positional difference between siRNA designed against mutant/wild-type allele. **3) *Gene information*** includes gene name, wild-type/mutant allele with mutated base/s highlighted. **4) *Respective gene/protein resources:*** hyperlinks to various gene/protein resources. **5) *Disease/mutation information*:** knowledge about mutation, type and its pathogenicity. **6) *Clinical resources:*** includes all comprehensive clinical/genetic knowledge collected from ClinVar, KEGG disease, COSMIC, DECIPHER, OMIM etc. **7) *ASP-siRNA* details:** includes ASP-siRNA, its efficacy for mutant/wild-type allele and their percent relative difference; while **8) *siRNA* targeting wild-type allele:** It deals with similar information in the context of *siRNA against wild-type allele*. **9) *General information*:** section harbors all the experimental details of the ASP-siRNA as mentioned in article **(Fig.S2-S5)**.

1. **Supplementary methods for Prediction algorithm section**
2. **Dataset preparation: ASPsiPred^SVM^**

In the first step, we started with total 4543 ASP-siRNA sequences and removed the 422 cm ASP-siRNAs, as our aim was to predict Eff^mut^ without any chemical modifications. Thereafter, we have screened 4121 ASP-siRNAs to extract unique, non-redundant ASP-siRNAs. Firstly, those entries in which mismatch information was not available (NA) were removed. Since we were developing a regression-based predictor, therefore those instances in which Effmut was either “NA” or “qualitative” (e.g. poor efficacy/moderate efficacy) were removed. There were some sequences having clash between the siRNA: mutant target, we have removed those sequences too (Du et al., 2005). Since, our focus was to develop the ASPsiPred having full complementarity with mutant allele (ASPsiPred^SVM^). However, in the database, there were 1136 sequences having mismatch/s with mutant allele. Cases of up to four mismatches have been reported in studies. We have only taken data of fully complementary ASP-siRNAs with mutant allele sequences. Next, we have extracted ASP-siRNAs of different lengths. 1545 sequences belong to 19mer length from the above dataset; while rest of the 310 sequences belongs to other than 19mer length. Removal of redundant sequences to get unique 19mer ASP-siRNAs resulted in the dataset of 922 ASP-siRNAs (D922) (**Table S2)**.

1. **Overlap in D922 dataset:**

Generally, ASP-siRNA is designed in such a way to inhibit mutant allele maximum with least inhibition of wild-type allele. Therefore, in order to find the optimum discriminatory ASP-siRNA, *the nucleotide complementary to mutation* (N^mutcomp^) can be placed from 1^st^ to 19^th^ position on siRNA. There are many experimental studies in which *‘*N^mutcomp^’ is placed at these 19 different positions of siRNA and its effect on efficacy has been demonstrated experimentally. Thus, these 19 ASP-siRNAs targeted against a single mutation are overlapped sequences having “N^mutcomp^” shifting forward with single nucleotide sliding window. Though, these sequences posess only single nucleotide change, but their efficacy is quite different at different positions (w.r.t N^mutcomp^). We have obtained D922 ASP-siRNAs dataset. It contains 17 such studies of 19 ASP-siRNA sequences. So, our database has many sequences having only single nucleotide change but they exhibit difference in efficacy. Many siRNA prediction algorithms are there but none of them have been exclusively trained on single nucleotide sliding trails.

1. **Features used for model development**:

Nucleotide composition refers to total number of a particular nucleotide divided by length of siRNA. In past, it is already reported that nucleotide composition is vital factor in predictive SVM models (Peek, 2007). We have computed mono-, di-, tri-, tetra- and penta-nucleotide composition of siRNA along with their hybrid combinations. For e.g. mono-and di- nucleotide composition generates a vector of 4 (A, C, G, and T) and 16 features (AA, AC, AG, CG,..., UU) respectively.

Position-specific feature corresponds to occupancy of a nucleotide at a particular position of the siRNA sequence (Huesken et al., 2005; McQuisten and Peek, 2009; Peek, 2007; Shabalina et al., 2006; Vert et al., 2006). This position specific occupancy is then converted to binary codes for each of the four nucleotides; i.e. A=1000, C=0100, G=0010, U=0001 to make it SVM readable format. Thus for a 19-mer siRNA, it resulted in generation of 76 patterns; also termed as “binary pattern of nucleotides”. It provides information about the relative preference of a nucleotide at each position of siRNA.

Since, thermodynamic stability and secondary structure of siRNA is critical parameters for siRNA designing (Shabalina et al., 2006). We have also computed secondary structure using RNAfold program of Vienna RNA package. In total 21 and 19 features were used to calculate thermodynamic property and secondary structure of a siRNA respectively. Vectors generated from above mentioned features were converted to binary format to make it SVM readable layout.

1. **Algorithm development**

In this study, we have used radial basis function (RBF) kernel for development of *ASPsiPred^SVM^*.


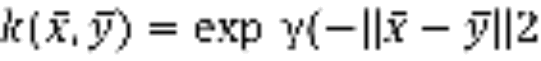


Where
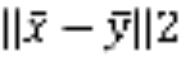
 is termed as squared Euclidean distance between two feature vectors x and y and ϒ is the tuning parameter and k is kernel function. ‘*Gamma*’ (**g OR** γ) and *Regularization parameter* ‘**c’** are the hyperparameters of RBF kernel of Support Vector Machines (SVM). Gamma (g) defines this separating hyperplane and C is the parameter for the soft margin cost function, which best classifies the training dataset either by hard or soft margin depending upon the dataset requirements.

1. **Validation**

All models were trained and tested using 10-fold cross validation (10nCV). In this process, dataset is randomly divided into ten sets, where one set termed as test set is tested by model developed on remaining nine sets (training sets) and the whole process is repeated ten times. We have evaluated the performance of our models on the basis of Pearson’s correlation coefficient (PCC), which is actually average of the PCC of ten training-testing steps. Apart from ten-fold cross-validation, we have also tested our models on independent validation dataset not used anywhere in training/testing process.


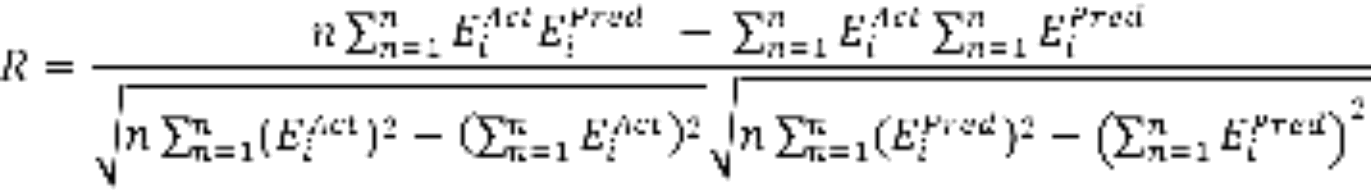


1. **Dataset preparation: ASPsiPred^matrix^**

These studies were designed to observe effect of residue clash between siRNA: mRNA at different 19 locations on Eff^wild^. We have used these findings to generate a matrix incorporating the effect of one-mismatch on siRNA efficacy. The approach to generate a 2D-matrix from different studies is summarized below:

1. We have assigned data in two parts: first, efficacy of siRNAs with perfect complementary siRNA: mRNA pair at 19 locations (for e.g. G:C^18^, A:U^16^ etc.) was extracted. Data regarding efficacy to silence fully complementary siRNA: mRNA pair (**Data*^Comp^***) at 19 possible locations has been taken as reference point (see **Table S5-S8 S2-S5**). For e.g. G:C match with Guanine at18^th^ position of guide strand (**G-18** siRNA) with fully complementary target sequence is symbolized by **G:C^18^**. All instances of **G-18** were compiled from above stated articles and their inhibition was averaged (as illustrated in **Table S2**). Similarly, average efficacy of siRNA: mRNA match at particular location was entitled as **Eff*^Match-Position-Avg^***; for e.g. average inhibition of **G-18** siRNA with perfect match **G:C^18^** from above mentioned studies was designated as **Eff^G:C-^*^18-^*^Avg^**.
2. In second portion, we collected the data pertaining to efficacy of siRNA having one clash with target sequence at 19 different locations (**Data*^Clash^***). Taking the example of G-18 siRNA, it can have three types of clash combinations with target mRNA i.e. G:A, G:G, G:U designated as G:A^18^, G:G^18^, G:U^18^ respectively (as illustrated in **Table S5 S2**). Accordingly, average efficacy to silence target with one siRNA: mRNA clash was designated as **Eff^G:A-^*^18-^*^Avg^, Eff^G:G-^*^18-^*^Avg^, Eff^G:U-^*^18-^*^Avg^** respectively. Thus, a mismatch discrimination profile for siRNA: mRNA pairs were generated keeping Guanine at 19 possible locations of siRNA guide strand (**Table S5 S2**). Similarly, discrimination profile was also built for other nucleotides than Guanine (**Table S6-S8 S3-S5**).

c) Finally, we have calculated the effect of siRNA: mRNA clash at a particular location on efficacy by subtracting the efficacy of clashed pair with that of perfect complementary pair, e.g. **Effect^G:A^_Clash =_ Eff^G:A-^*^18-^*^Avg^- Eff^G:C-^*^18-^*^Avg^ -** i.e. 87-86=1 (**Table S5 S2**). Following this approach, we have constructed four matrices. Thus, resultant matrix is constructed for four nucleotides with prefect and mismatched pair at 19 possible locations and integrated on webserver.

**Supplementary Tables**

**Table S1. List of computational resources for experimentally validated siRNAs**

| **S.No** | **Database** | **Description of sequences** | **No. of entries** | **Organism** | **ASP-siRNA sequences** | **Reference** |
| --- | --- | --- | --- | --- | --- | --- |
| 1 | HuSiDa | Human siRNAs | 1158 | Human | **✕** | (Truss et al., 2005) |
| 2 | siRecords | Mammalian siRNAs | 17,192 | Mammalian | **✕** | 16443930, 18996894 |
| 3 | HIVsirDB | HIV Inhibiting siRNAs | 750 | HIV | **✕** | (Tyagi et al., 2011) |
| 4 | VirsiRNAdb | Viral siRNAs | 1358 | Virus | **✕** | (Thakur et al., 2012) |
| 5 | RNAiAtlas | siRNA libraries and their specificity | 1,96,024 | Human | **✕** | (Mazur et al., 2012) |
| 6 | GenomeRNAi | Cell-based and in vivo RNAi phenotypes | 5,00,000 phenotypes | Human | **✕** | (Schmidt et al., 2013) |
| 7 | EHFPI | Essential host factors (EHFs) from genome-wide RNAi screens | 4634 EHFs | Human | **✕** | (Liu et al., 2015) |
| 8 | siRNAmod | Chemically modified siRNAs (cm-siRNAs) | 4894 | Mammalian | **✕** | 26818131 |
| 9 | ASPsiRNA | Allele specific siRNAs database and efficacy prediction algorithm | 4543 | Human | **✓** | <http://crdd.osdd.net/servers/aspsirna/> |

**Table S2. Steps involved in the extraction of 922 ASP-siRNAs dataset for ASPsiPred^SVM^**

| **S.No.** | **Step performed** | **Sequences Removed** | **Remaining sequences** |
| --- | --- | --- | --- |
| 1 | Initial dataset taken | 4543 | |
| 2 | Removal of chemical modified ASP-siRNAs | 422 | 4121 |
| 3 | Removed entries in which mismatch information was not available | 842 | 3279 |
| 4 | Removed sequences in which Eff^mut^ was NA | 212 | 3067 |
| 5 | Removed qualitative Eff^mut^ | 17 | 3050 |
| 6 | Studies having clash between the siRNA: mutant target (since, model developed to predict the fully complementary siRNA efficacy) | 59 | 2991 |
| 7 | Sequences with mismatches with mutant allele | 1136 | 1855 |
| 8 | 19mer ASP-siRNA sequences | 310 | 1545 |
| 9 | Removal of redundant sequences | 623 | 922 |
| 10 | Dataset of non-redundant ASP-siRNA sequences | 922 | |

**Table S3. Performance of random independent validation sets (V185) using SVM models developed on their respective training/testing sets (T737) employing 10-fold cross-validation (10nCV)**

|  | **Parameters used (RBF kernel)** | | **PCC on training/testing sets (T737) and independent validation sets (V185) using 10nCV** | |
| --- | --- | --- | --- | --- |
| **S.No.** | **g** | **c** | **T737** | **V185** |
| Random Set-1 | 0.001 | 200 | 0.72 | 0.63 |
| Random Set-2 | 0.001 | 50 | 0.71 | 0.65 |
| Random Set-3 | 0.001 | 50 | 0.71 | 0.63 |
| Random Set-4 | 0.001 | 100 | 0.70 | 0.69 |
| Random Set-5 | 0.001 | 50 | 0.69 | 0.69 |

**Table S4. Results of 10-fold cross-validation (10nCV) on complete dataset T922**

| **No.** | **siRNA feature name** | **No. of features** | **PCC** |
| --- | --- | --- | --- |
| 1 | Mononucleotide composition | 4 | 0.53 |
| 2 | Dinucleotide composition | 16 | 0.68 |
| 3 | Trinucleotide composition | 64 | 0.71 |
| 4 | Tetranucleotide composition | 256 | 0.69 |
| 5 | Pentanucleotide composition | 1024 | 0.70 |
| 6 | Binary | 76 | 0.57 |
| 7 | 1+2 | 20 | 0.66 |
| 8 | 1+2+3 | 84 | 0.70 |
| 9 | 1+2+3+4 | 340 | 0.71 |
| 10 | 1+2+3+4+5 | 1364 | 0.71 |
| 11 | 1+2+3+4+6  **(ASPsiPred^SVM#^)** | 416 | 0.71 |
| 12 | 1+2+3+4+5+6 | 1440 | 0.71 |

**Table S5.** Mismatch discrimination profile made from rule-based studies for Guanine (G) at 19 possible locations of siRNA guide strand. This profile for Guanine was utilized in developing final matrix i.e. ASPsiPred^matrix^

| **Efficacy of target mRNA** | | | | | |  |  |  |  |
| --- | --- | --- | --- | --- | --- | --- | --- | --- | --- |
| **Guide strand** | **mRNA** | **A** | **C** | **G** | **U** | **G:A clash** | **G:C perfect pair** | **G:G clash** | **G:U clash** |
|  | | **Eff^G:A Avg^** | **Eff^G:C Avg^** | **Eff^G:G Avg^** | **Eff^G:U Avg^** | **Effect^G:A Clash^** | **Effect^G:C Pair^** | **Effect^G:G Clash^** | **Effect^G:U Clash^** |
| **G1-avg** | | 92 | 91 | 92 | 92 | 1 | 0 | 1 | 1 |
| **G2-avg** | | 58 | 90 | 68 | 69 | -32 | 0 | -22 | -21 |
| **G3-avg** | | 17 | 87 | 30 | 57 | -70 | 0 | -57 | -30 |
| **G4-avg** | | 67 | 92 | 66 | 80 | -26 | 0 | -27 | -13 |
| **G5-avg** | | 28 | 90 | 45 | 57 | -62 | 0 | -45 | -33 |
| **G6-avg** | | 63 | 92 | 60 | 32 | -30 | 0 | -32 | -60 |
| **G7-avg** | | 81 | 90 | 68 | 84 | -9 | 0 | -23 | -7 |
| **G8-avg** | | 39 | 87 | 47 | 71 | -48 | 0 | -40 | -16 |
| **G9-avg** | | 23 | 90 | 33 | 65 | -67 | 0 | -57 | -25 |
| **G10-avg** | | 50 | 92 | 40 | 64 | -42 | 0 | -52 | -28 |
| **G11-avg** | | 38 | 91 | 40 | 44 | -53 | 0 | -51 | -47 |
| **G12-avg** | | 50 | 87 | 32 | 55 | -37 | 0 | -55 | -32 |
| **G13-avg** | | 40 | 90 | 42 | 45 | -50 | 0 | -49 | -45 |
| **G14-avg** | | 64 | 87 | 70 | 79 | -23 | 0 | -16 | -8 |
| **G15-avg** | | 83 | 87 | 46 | 70 | -5 | 0 | -41 | -18 |
| **G16-avg** | | 62 | 92 | 68 | 64 | -30 | 0 | -25 | -29 |
| **G17-avg** | | 94 | 96 | 91 | 95 | -2 | 0 | -4 | -1 |
| **G18-avg** | | 87 | 86 | 86 | 88 | 1 | 0 | 0 | 2 |
| **G19-avg** | | 90 | 90 | 97 | 95 | 0 | 0 | 7 | 5 |

**Table S6.** Mismatch discrimination profile constructed from different studies for Adenine (A) at 19 possible locations of siRNA guide strand

| **Efficacy of target mRNA** | | | | | |  |  |  |  |
| --- | --- | --- | --- | --- | --- | --- | --- | --- | --- |
| **Guide strand** | **mRNA** | **A** | **C** | **G** | **U** | **A:A clash** | **A:C clash** | **A:G clash** | **A:U perfect pair** |
|  | | **Eff^G:A Avg^** | **Eff^A:A Avg^** | **Eff^A:C Avg^** | **Eff^A:G Avg^** | **Eff^A:U Avg^** | **Effect^A:A Clash^** | **Effect^A:G Clash^** | **Effect^A:C Pair^** |
| **A1-avg** | | 89 | 79 | 77 | 88 | 1 | -9 | -11 | 0 |
| **A2-avg** | | 87 | 79 | 64 | 90 | -3 | -11 | -26 | 0 |
| **A3-avg** | | 45 | 61 | 50 | 86 | -41 | -25 | -36 | 0 |
| **A4-avg** | | 70 | 74 | 68 | 91 | -21 | -17 | -24 | 0 |
| **A5-avg** | | 75 | 84 | 63 | 90 | -16 | -6 | -28 | 0 |
| **A6-avg** | | 73 | 79 | 77 | 90 | -18 | -11 | -14 | 0 |
| **A7-avg** | | 60 | 73 | 73 | 90 | -30 | -17 | -17 | 0 |
| **A8-avg** | | 50 | 66 | 64 | 86 | -36 | -20 | -23 | 0 |
| **A9-avg** | | 52 | 55 | 50 | 90 | -38 | -35 | -40 | 0 |
| **A10-avg** | | 17 | 63 | 21 | 88 | -71 | -25 | -67 | 0 |
| **A11-avg** | | 18 | 18 | 17 | 87 | -69 | -69 | -70 | 0 |
| **A12-avg** | | 91 | 66 | 67 | 92 | -1 | -26 | -25 | 0 |
| **A13-avg** | | 68 | 65 | 44 | 86 | -18 | -21 | -42 | 0 |
| **A14-avg** | | 25 | 44 | 13 | 90 | -65 | -46 | -77 | 0 |
| **A15-avg** | | 50 | 81 | 50 | 90 | -40 | -9 | -40 | 0 |
| **A16-avg** | | 42 | 62 | 45 | 93 | -51 | -31 | -48 | 0 |
| **A17-avg** | | 72 | 82 | 70 | 90 | -18 | -8 | -20 | 0 |
| **A18-avg** | | 83 | 85 | 85 | 90 | -7 | -5 | -5 | 0 |
| **A19-avg** | | 82 | 88 | 92 | 90 | -8 | -2 | 2 | 0 |

**Table S7.** Mismatch discrimination profile made from rule-based studies for Uracil (U) at 19 possible locations of siRNA guide strand

| **Efficacy of target mRNA** | | | | | |  |  |  |  |
| --- | --- | --- | --- | --- | --- | --- | --- | --- | --- |
| **Guide strand** | **mRNA** | **A** | **C** | **G** | **U** | **U:A perfect pair** | **U:C clash** | **U:G clash** | **U:U clash** |
|  | | **Eff^U:A Avg^** | **Eff^U:C Avg^** | **Eff^U:G Avg^** | **Eff^U:U Avg^** | **Effect^U:A Pair^** | **Effect^U:C Clash^** | **Effect^U:G Clash^** | **Effect^U:U Clash^** |
| **U1-avg** | | 89 | 75 | 73 | 84 | 0 | -14 | -16 | -5 |
| **U2-avg** | | 86 | 74 | 63 | 67 | 0 | -12 | -23 | -19 |
| **U3-avg** | | 90 | 51 | 78 | 78 | 0 | -39 | -12 | -12 |
| **U4-avg** | | 87 | 66 | 64 | 74 | 0 | -21 | -23 | -12 |
| **U5-avg** | | 86 | 68 | 71 | 82 | 0 | -18 | -15 | -4 |
| **U6-avg** | | 82 | 54 | 60 | 58 | 0 | -28 | -22 | -24 |
| **U7-avg** | | 86 | 59 | 73 | 81 | 0 | -27 | -13 | -5 |
| **U8-avg** | | 83 | 38 | 80 | 58 | 0 | -45 | -3 | -25 |
| **U9-avg** | | 86 | 20 | 67 | 5 | 0 | -66 | -19 | -81 |
| **U10-avg** | | 87 | 59 | 39 | 50 | 0 | -29 | -48 | -37 |
| **U11-avg** | | 87 | 27 | 52 | 15 | 0 | -60 | -35 | -72 |
| **U12-avg** | | 94 | 49 | 74 | 50 | 0 | -45 | -19 | -44 |
| **U13-avg** | | 82 | 30 | 32 | 45 | 0 | -52 | -50 | -37 |
| **U14-avg** | | 88 | 45 | 63 | 85 | 0 | -44 | -25 | -3 |
| **U15-avg** | | 90 | 57 | 66 | 43 | 0 | -34 | -24 | -48 |
| **U16-avg** | | 86 | 55 | 60 | 69 | 0 | -31 | -27 | -18 |
| **U17-avg** | | 92 | 84 | 78 | 88 | 0 | -7 | -14 | -3 |
| **U18-avg** | | 84 | 49 | 60 | 77 | 0 | -35 | -24 | -7 |
| **U19-avg** | | 90 | 95 | 94 | 95 | 0 | 5 | 4 | 5 |

**Table S8.** Mismatch discrimination profile built from rule-based studies for Cytosine (C) at 19 possible locations of siRNA guide strand

| **Efficacy of target mRNA** | | | | | |  |  |  |  |
| --- | --- | --- | --- | --- | --- | --- | --- | --- | --- |
| **Guide strand** | **mRNA** | **A** | **C** | **G** | **U** | **C:A clash** | **C:C clash** | **C:G perfect pair** | **C:U clash** |
|  | | Eff^C:A Avg^ | Eff^C:C Avg^ | Eff^C:G Avg^ | Eff^C:U Avg^ | Effect^C:A Clash^ | Effect^C:C Clash^ | Effect^C:G Pair^ | Effect^C:U Clash^ |
| **C1-avg** | | 90 | 89 | 90 | 90 | 0 | -1 | 0 | 0 |
| **C2-avg** | | 82 | 81 | 84 | 82 | -2 | -3 | 0 | -2 |
| **C3-avg** | | 83 | 75 | 90 | 75 | -7 | -15 | 0 | -15 |
| **C4-avg** | | 85 | 70 | 94 | 82 | -10 | -24 | 0 | -12 |
| **C5-avg** | | 83 | 83 | 88 | 74 | -5 | -5 | 0 | -14 |
| **C6-avg** | | 88 | 68 | 90 | 72 | -3 | -23 | 0 | -18 |
| **C7-avg** | | 87 | 73 | 87 | 75 | 0 | -14 | 0 | -12 |
| **C8-avg** | | 74 | 58 | 90 | 71 | -17 | -32 | 0 | -19 |
| **C9-avg** | | 61 | 50 | 91 | 63 | -30 | -41 | 0 | -28 |
| **C10-avg** | | 55 | 30 | 87 | 52 | -32 | -57 | 0 | -36 |
| **C11-avg** | | 73 | 58 | 90 | 47 | -17 | -32 | 0 | -43 |
| **C12-avg** | | 72 | 29 | 91 | 61 | -18 | -62 | 0 | -30 |
| **C13-avg** | | 82 | 50 | 90 | 55 | -8 | -40 | 0 | -35 |
| **C14-avg** | | 79 | 52 | 86 | 62 | -7 | -34 | 0 | -24 |
| **C15-avg** | | 92 | 70 | 94 | 88 | -2 | -24 | 0 | -6 |
| **C16-avg** | | 57 | 15 | 92 | 35 | -35 | -77 | 0 | -57 |
| **C17-avg** | | 91 | 81 | 93 | 87 | -3 | -13 | 0 | -7 |
| **C18-avg** | | 83 | 77.5 | 87.5 | 81 | -5 | -10 | 0 | -7 |
| **C19-avg** | | NA | NA | NA | NA | NA | NA | NA | NA |

**Table S9.** **Overall comparison of ASPsiPred^matrix^ and desiRm using four different experimental studies having nucleotide complementary to mutation on across 19mer ASP-siRNA**

[Note: Performance is measured by PCC between predicted and actual inhibitory efficacy of ASP-siRNA for wild-type allele with one mismatch (Eff^wild^)]

|  |  | **Performance of both methods to predict Eff^wild^ in Pearson Correlation Coefficient (PCC)** | |
| --- | --- | --- | --- |
| **S.No.** | **Reference** | **ASPsiPred^matrix^** | **desiRm** |
| 1 | (Liao et al., 2011) | 0.49 | 0.08 |
| 2 | (Atkinson et al., 2011) | 0.52 | 0.34 |
| 3 | (Courtney et al., 2014) | 0.46 | 0.02 |
| 4 | (Barbaro et al., 2016) | 0.35 | 0.15 |

**Table S10. Descriptive statistics used during histogram, normal density distribution curve, skewness and kurtosis calculation**

| **S.No.** | **Descriptive** | **Statistic** |
| --- | --- | --- |
| 1 | Mean | 61.24 |
| 2 | Median | 70 |
| 3 | Mode | 90 |
| 4 | Std. deviation | 29.14 |
| 5 | Variance | 849.34 |
| 6 | Maximum | 100 |
| 7 | Minimum | 0 |
| 8 | Range | 100 |
| 9 | Skewness | -0.55 |
| 10 | Kurtosis | -0.89 |

**Supplementary Figures**

**
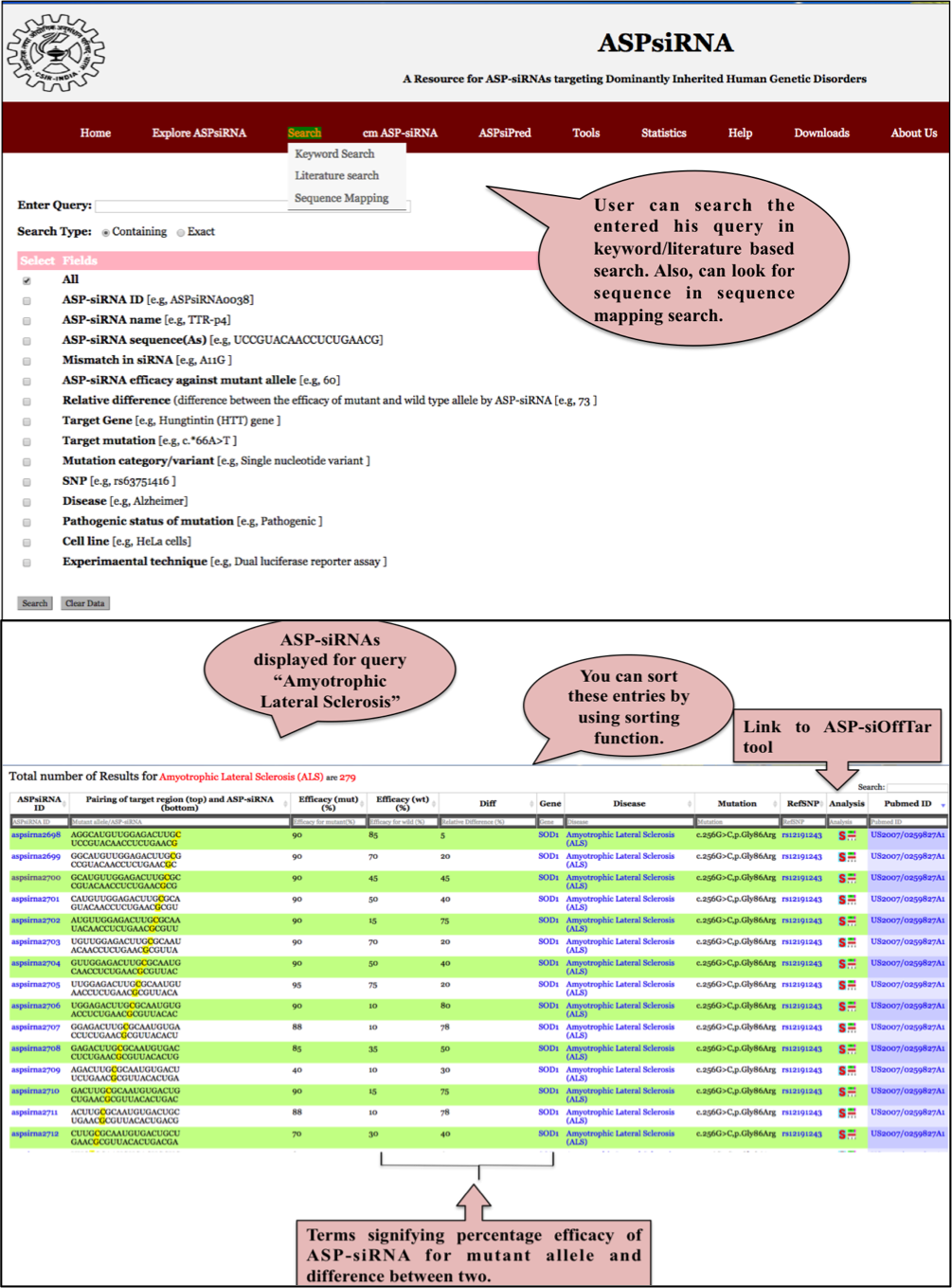
**

**Figure S1.** An example screenshot representing the search result for ASP-siRNAs with keyword “amyloid lateral sclerosis”


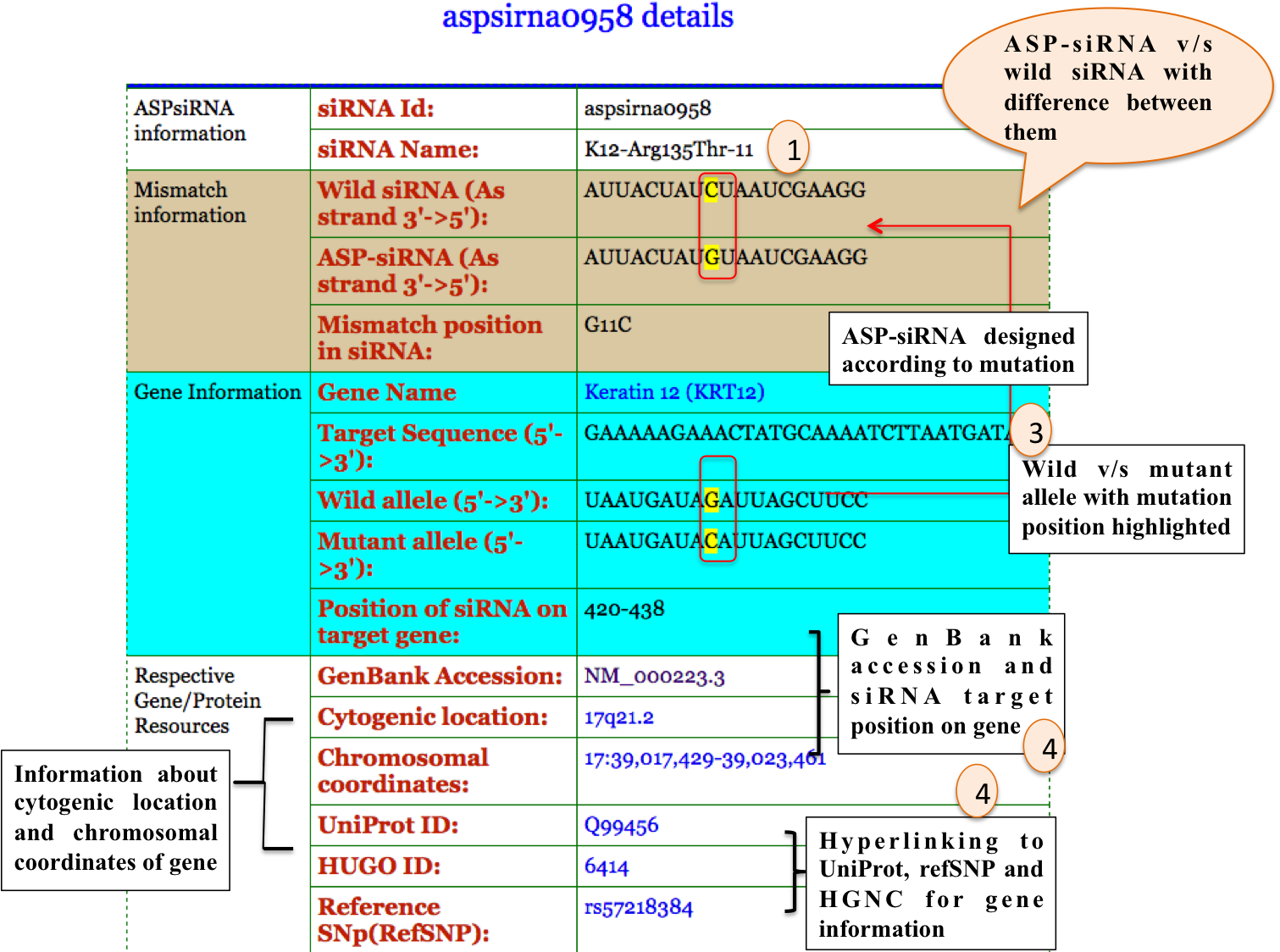


**Figure S2.** Screenshot demonstrating the first part of individual ASP-siRNAs record (this section harbors information on ASPsiRNA designed against mutant allele, location of mismatch on siRNA guide strand, gene and mutation location)


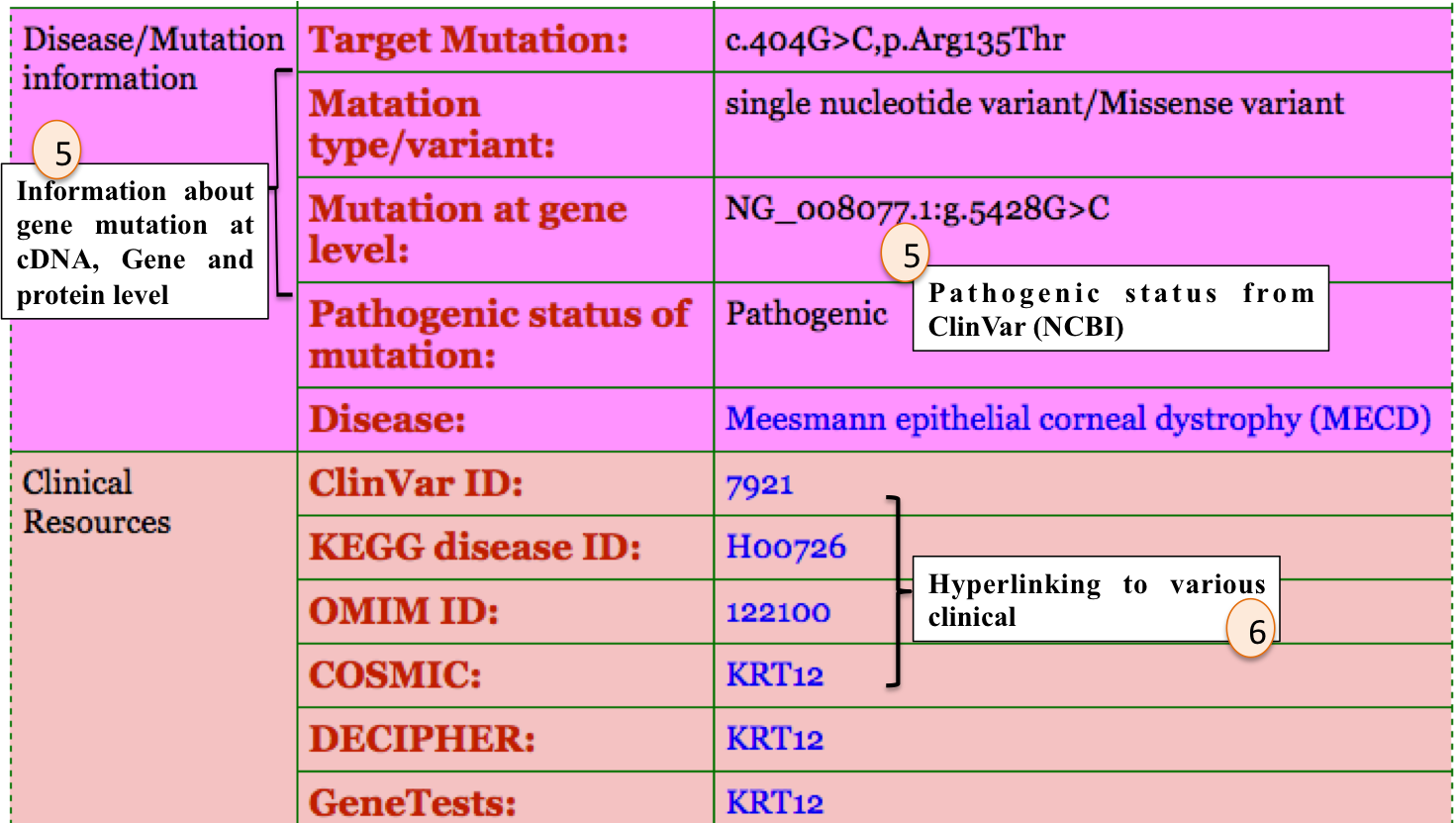


**Figure S3.** Screenshot demonstrating the second section of individual ASP-siRNAs entry, which entails data on various aspects of disease like pathogenicity, mutation in HGNC format and links to clinical resources


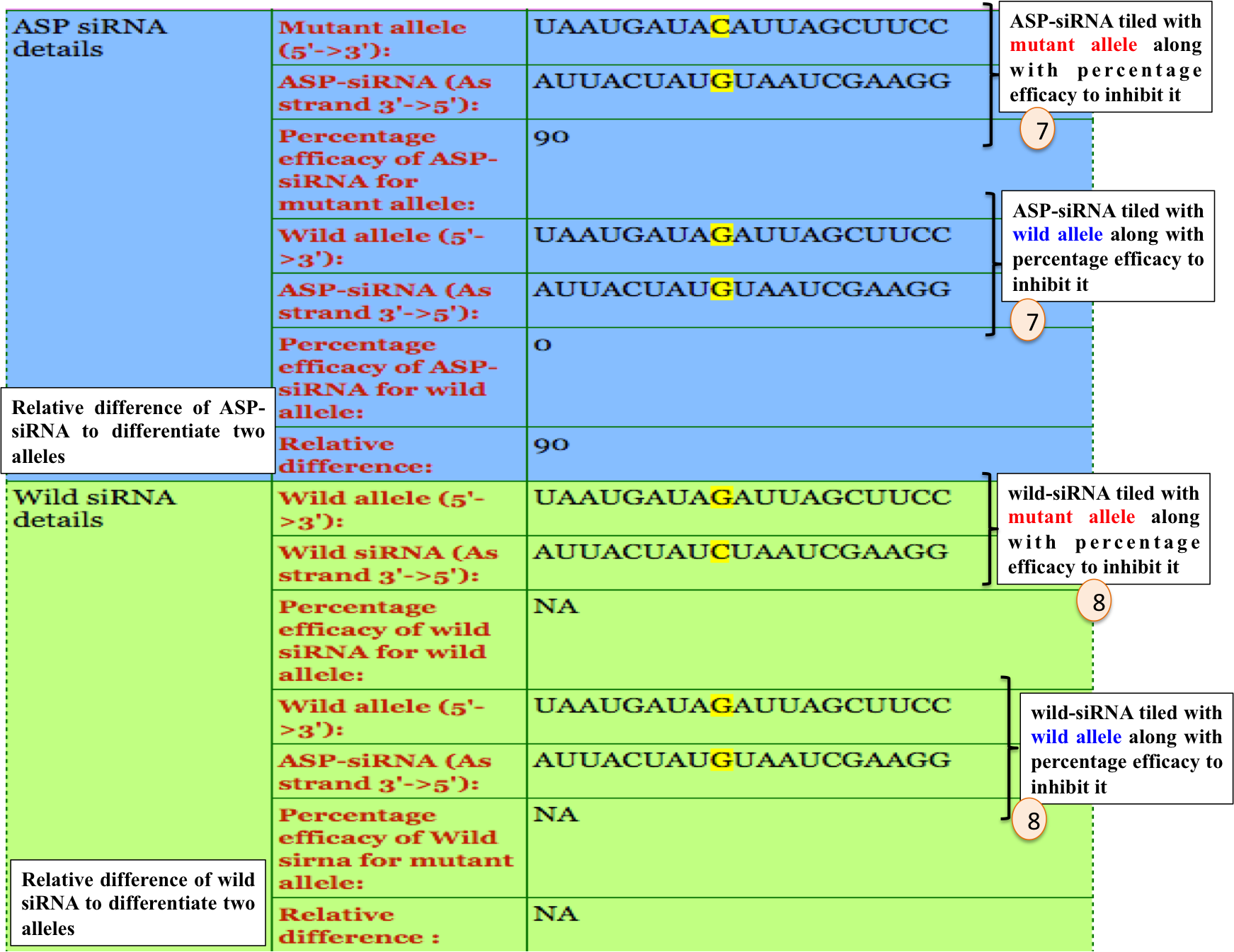


**Figure S4.** Figure representing the third section of individual record (this part is offering experimental data on Eff^mut^ and Eff^wild^ by ASP-siRNA and siRNA targeting wild-type allele)


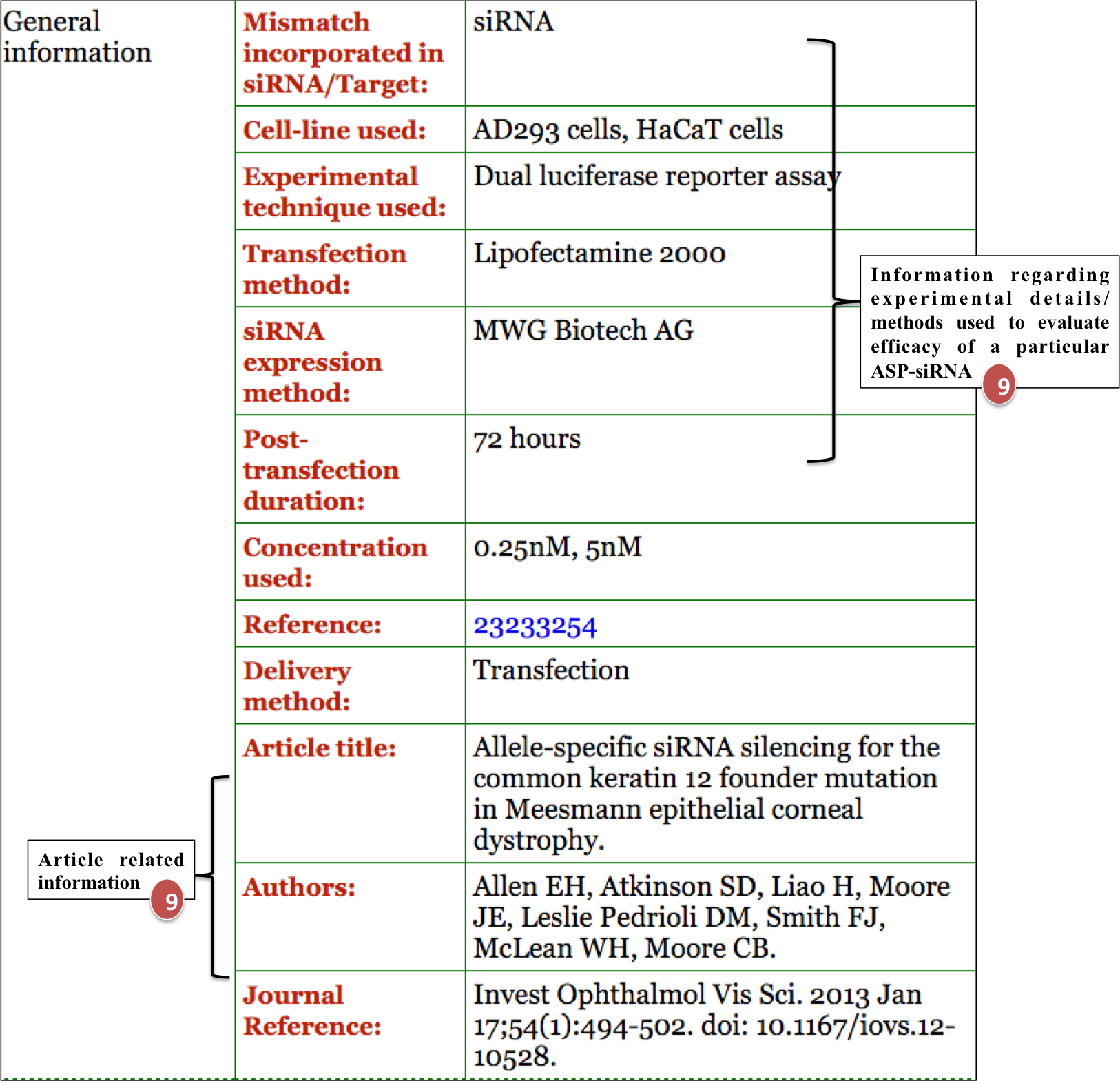


**Figure S5.** Figure demonstrating the fourth part of information obtained for individual ASP-siRNA entry


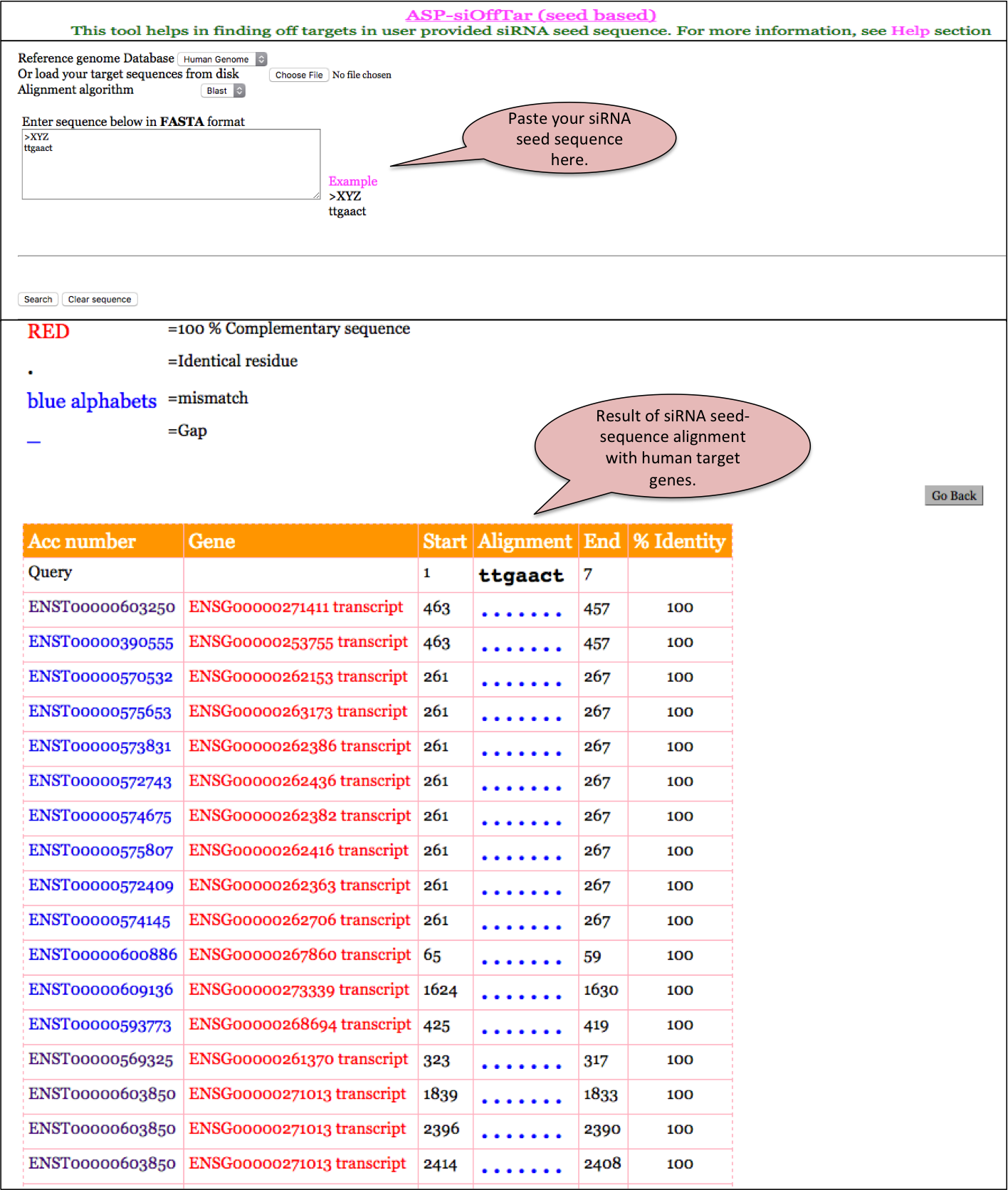


**Figure S6.** Screenshot representing the search result for seed based ASP-siOffTar tool

[**Note:** as shown in screenshot, Ensembl ID of aligned target sequence is provided along with start and end position. No mismatch is allowed in seed-based off-targets alignment on human genome]


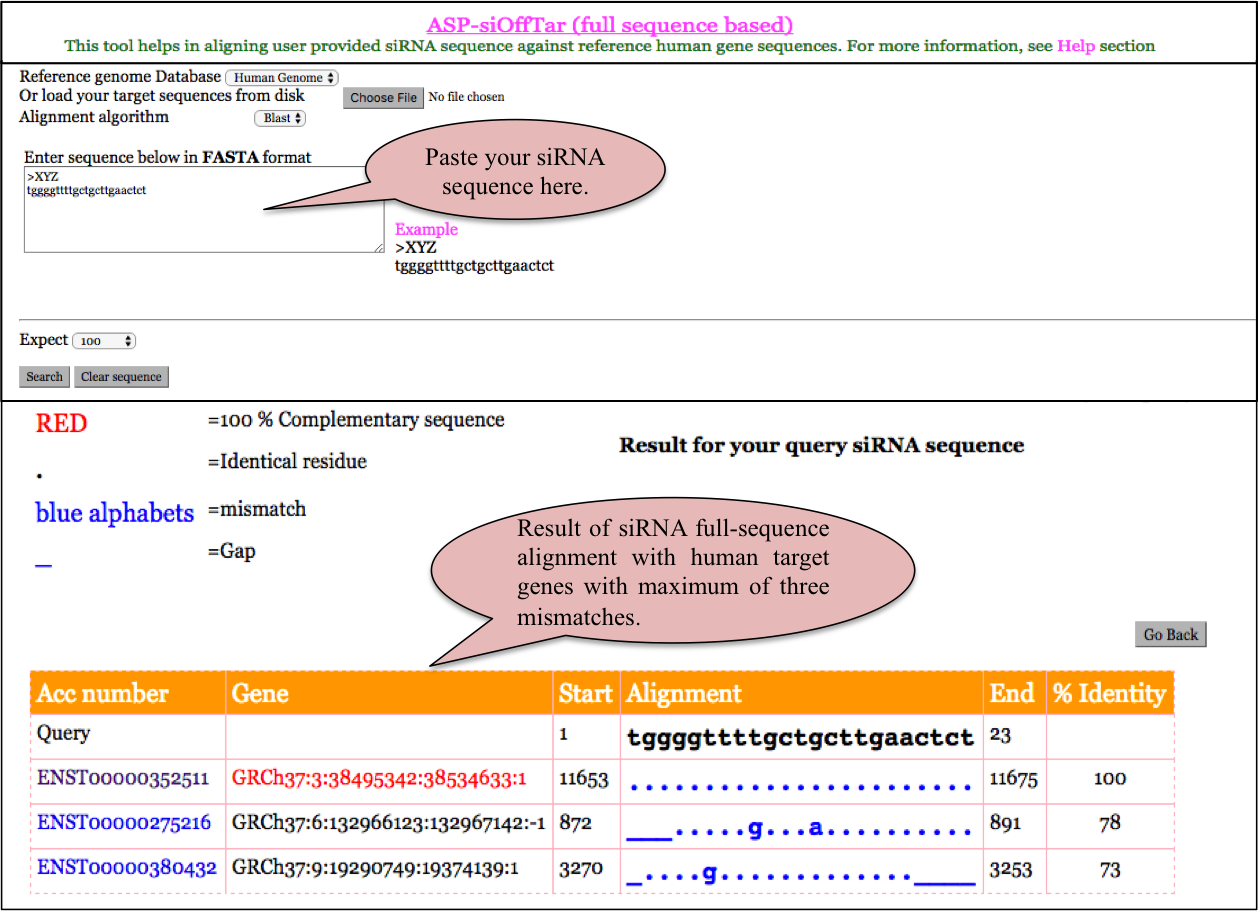


**Figure S7.** Screenshot representing the search result for full sequence based ASP-siOffTar tool

[**Note:** as shown in screenshot, Ensembl ID of aligned target sequence is provided along with start and end position. A maximum of three mismatches are allowed in off-targets based on alignment of full siRNA sequence on human genome]


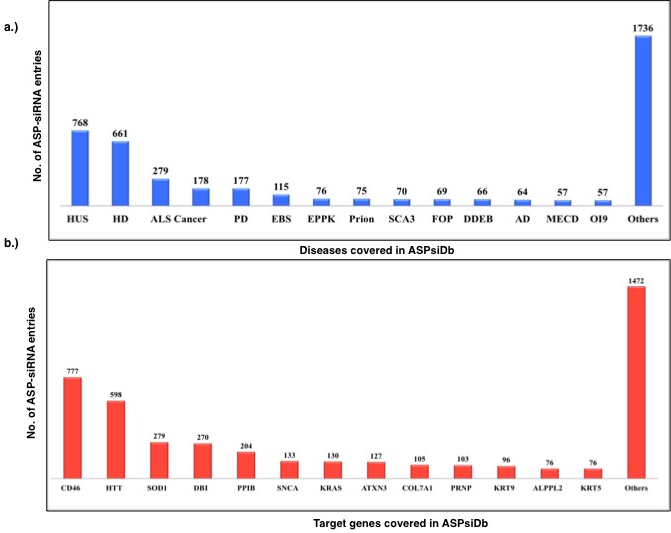


**Figure S8.** Bar graph depicting the statistical distribution of ASP-siRNAs for numerous human genetic diseases (a) and genes (b) reported in ASPsiDb (x-axis denotes human genes while y-axis signifies number of ASP-siRNAs)


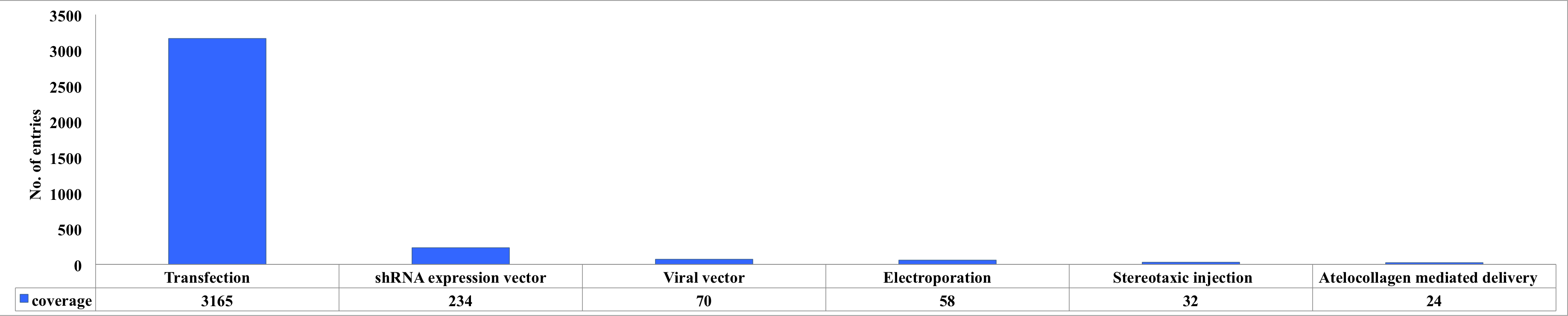
**Figure S9.** Bar graph representing the distribution of different delivery methods reported in ASPsiDb (x-axis denotes various delivery methods while y-axis signifies number of ASP-siRNAs)

**Figure S10.** A Smooth scatter plot in R displaying percentage efficacy distribution of ASP-siRNAs for mutant (Eff^mut^) versus wild-type allele (Eff^wild^) of target gene (x-axis represents efficacy for mutant allele, while y-axis signifies efficacy for wild-type allele)


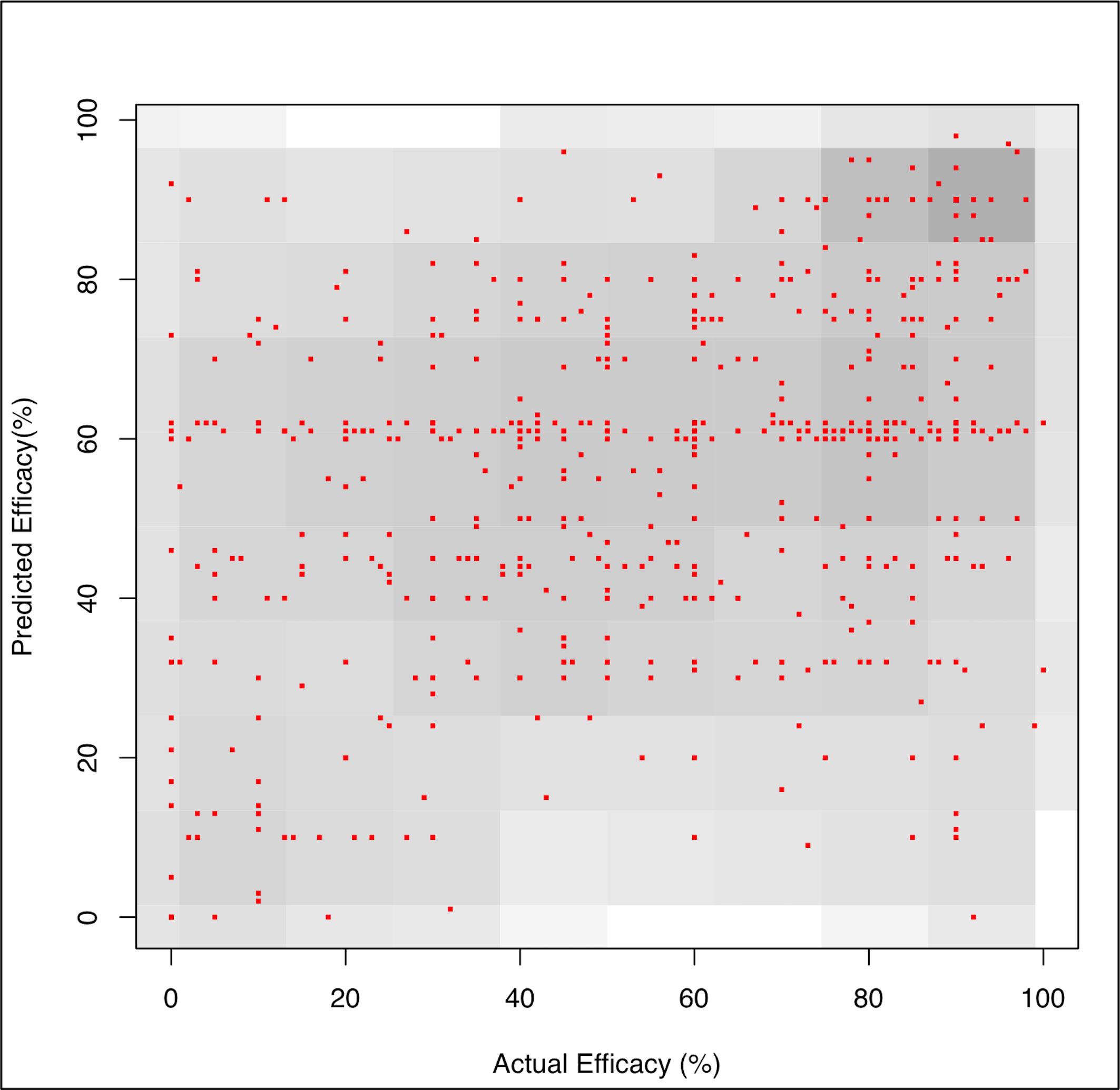


**Figure S11.** Smooth scatter plot in R depicting correlation between the actual and predicted percentage efficacy of T737 during 10nCV using SVM

**[Note-** T737: train/test set; 10nCV: ten-fold cross-validation**]**


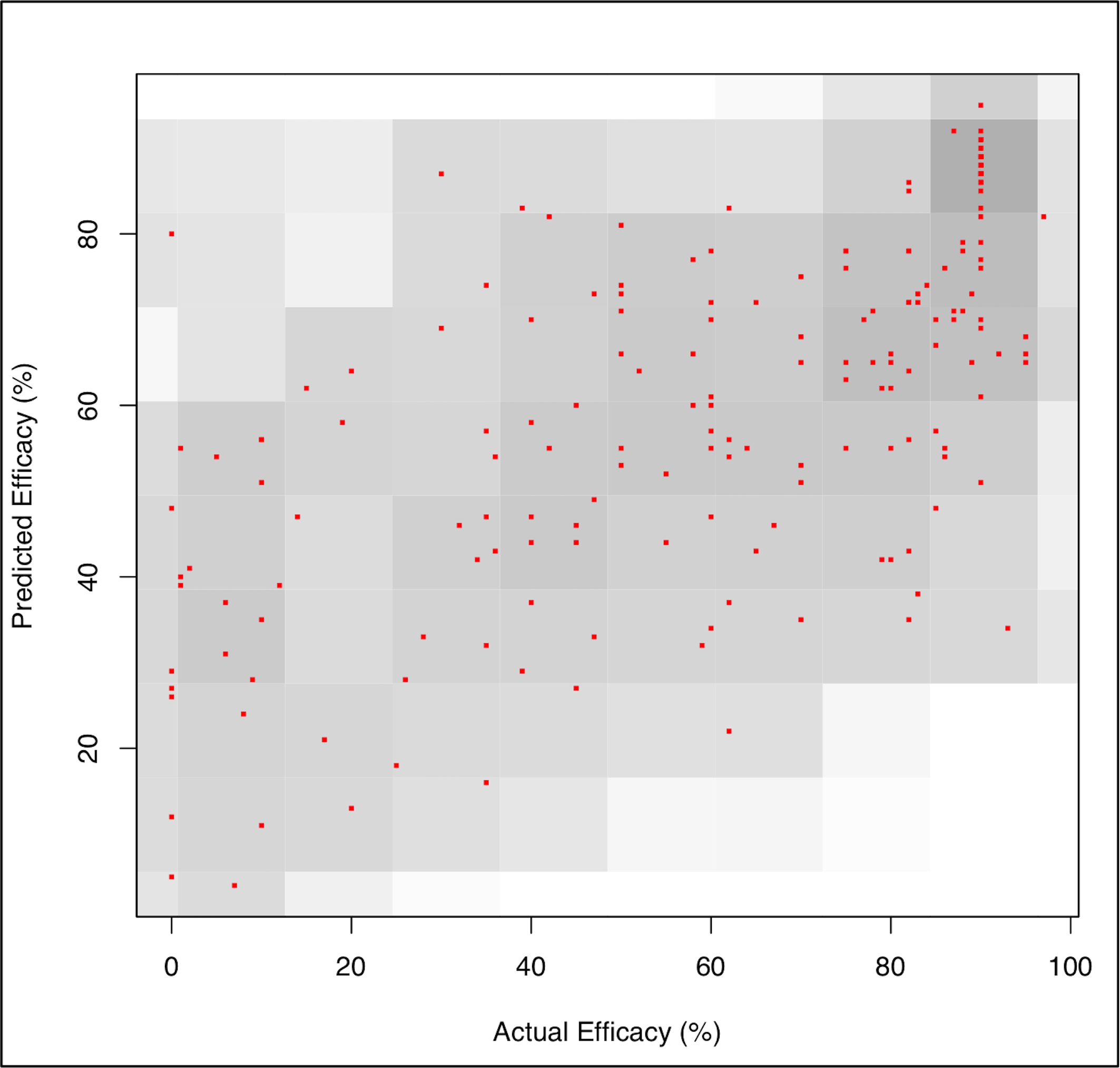
**Figure S12.** Smooth scatter plot in R illustrating correlation between the actual and predicted percentage efficacy of V185 during 10nCV using SVM

**[Note:** V185: validation set; 10nCV: ten-fold cross-validation**]**


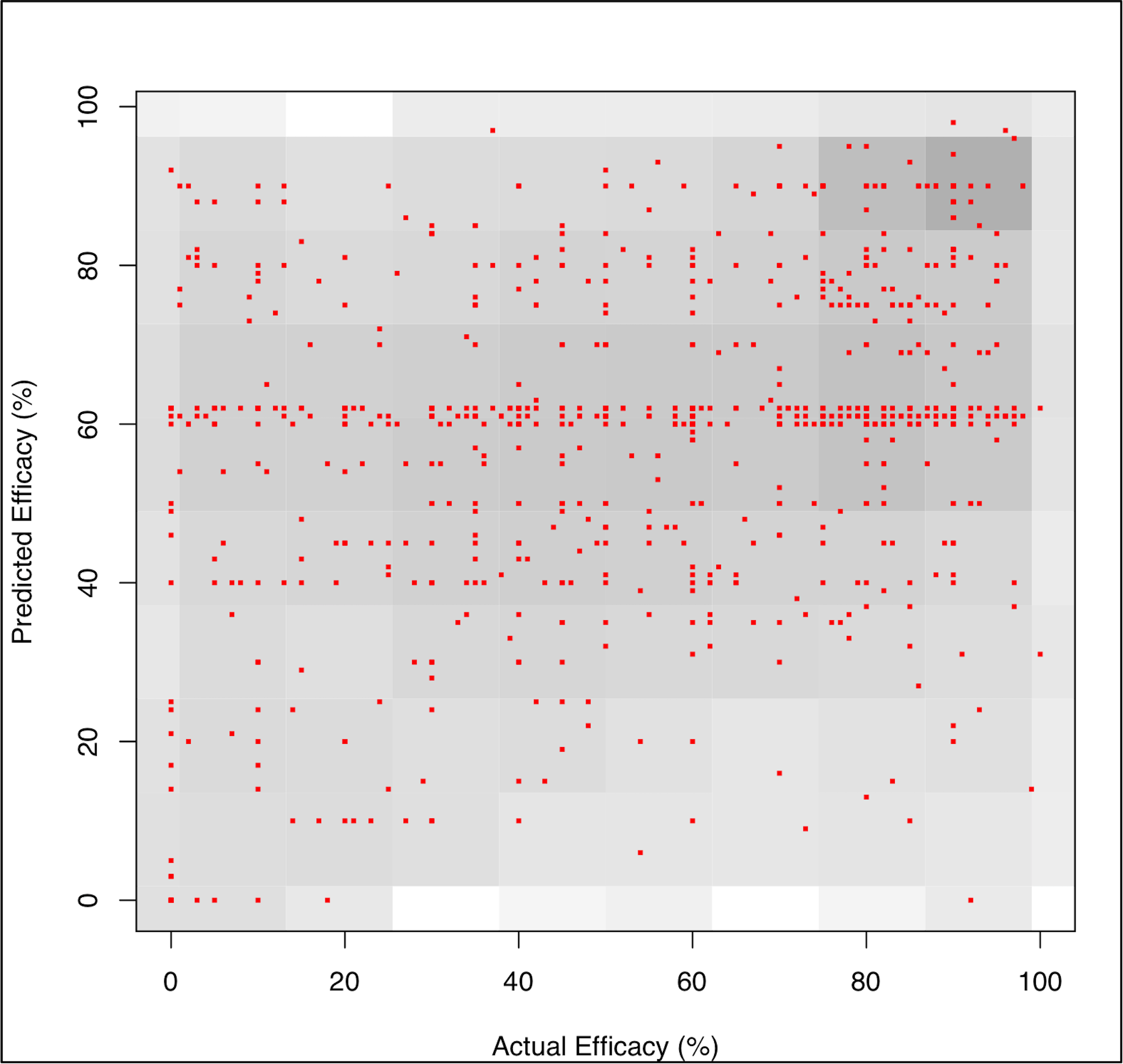
**Figure S13.** Smooth scatter plot in R representing correlation between the actual and predicted percentage efficacy of D922 during 10nCV using SVM

**[Note:** D922- Total ASP-siRNA dataset; 10nCV-ten-fold cross-validation**]**


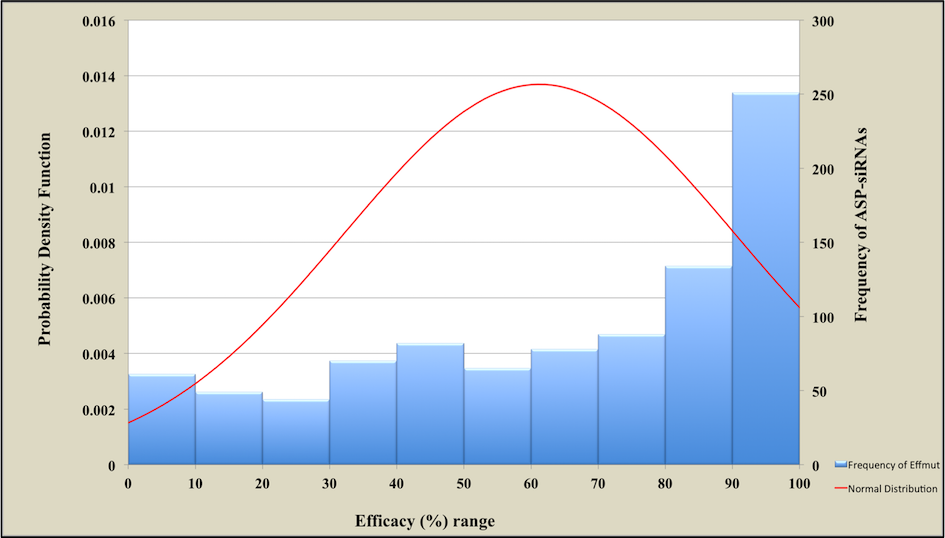
**Figure S14. Histogram with normal distribution function curve**: Histogram illustrating percent efficacy range (x-axis) and frequency of total ASP-siRNA dataset (D922) (z-axis). Normal density distribution curve depicting bell shaped curve with slightly right skew between percent efficacy (x-axis) and normal density for each efficacy data point using normal density distribution function (y-axis)

**Figure S15. Normality testing/outlier analysis by Z-value:** scatter plot representing Z-value versus expected value (red line) and z-value versus originals (blue line)

[Note: Expected value is calculated from CDF (cumulative distribution factor), mean and std. deviation]

**References**

Atkinson, S.D., McGilligan, V.E., Liao, H., Szeverenyi, I., Smith, F.J., Moore, C.B., and McLean, W.H. (2011). Development of allele-specific therapeutic siRNA for keratin 5 mutations in epidermolysis bullosa simplex. J Invest Dermatol *131*, 2079-2086.

Barbaro, V., Nasti, A.A., Del Vecchio, C., Ferrari, S., Migliorati, A., Raffa, P., Lariccia, V., Nespeca, P., Biasolo, M., Willoughby, C.E.*, et al.* (2016). Correction of Mutant p63 in EEC Syndrome Using siRNA Mediated Allele-Specific Silencing Restores Defective Stem Cell Function. Stem Cells *34*, 1588-1600.

Courtney, D.G., Atkinson, S.D., Moore, J.E., Maurizi, E., Serafini, C., Pellegrini, G., Black, G.C., Manson, F.D., Yam, G.H., Macewen, C.J.*, et al.* (2014). Development of allele-specific gene-silencing siRNAs for TGFBI Arg124Cys in lattice corneal dystrophy type I. Invest Ophthalmol Vis Sci *55*, 977-985.

Du, Q., Thonberg, H., Wang, J., Wahlestedt, C., and Liang, Z. (2005). A systematic analysis of the silencing effects of an active siRNA at all single-nucleotide mismatched target sites. Nucleic Acids Res *33*, 1671-1677.

Huesken, D., Lange, J., Mickanin, C., Weiler, J., Asselbergs, F., Warner, J., Meloon, B., Engel, S., Rosenberg, A., Cohen, D.*, et al.* (2005). Design of a genome-wide siRNA library using an artificial neural network. Nat Biotechnol *23*, 995-1001.

Liao, H., Irvine, A.D., Macewen, C.J., Weed, K.H., Porter, L., Corden, L.D., Gibson, A.B., Moore, J.E., Smith, F.J., McLean, W.H.*, et al.* (2011). Development of allele-specific therapeutic siRNA in Meesmann epithelial corneal dystrophy. PLoS One *6*, e28582.

Liu, Y., Xie, D., Han, L., Bai, H., Li, F., Wang, S., and Bo, X. (2015). EHFPI: a database and analysis resource of essential host factors for pathogenic infection. Nucleic Acids Res *43*, D946-955.

Lombardi, M.S., Jaspers, L., Spronkmans, C., Gellera, C., Taroni, F., Di Maria, E., Donato, S.D., and Kaemmerer, W.F. (2009). A majority of Huntington's disease patients may be treatable by individualized allele-specific RNA interference. Exp Neurol *217*, 312-319.

Loy, R.E., Lueck, J.D., Mostajo-Radji, M.A., Carrell, E.M., and Dirksen, R.T. (2012). Allele-specific gene silencing in two mouse models of autosomal dominant skeletal myopathy. PLoS One *7*, e49757.

Mazur, S., Csucs, G., and Kozak, K. (2012). RNAiAtlas: a database for RNAi (siRNA) libraries and their specificity. Database (Oxford) *2012*, bas027.

McQuisten, K.A., and Peek, A.S. (2009). Comparing artificial neural networks, general linear models and support vector machines in building predictive models for small interfering RNAs. PLoS One *4*, e7522.

Miller, V.M., Gouvion, C.M., Davidson, B.L., and Paulson, H.L. (2004). Targeting Alzheimer's disease genes with RNA interference: an efficient strategy for silencing mutant alleles. Nucleic Acids Res *32*, 661-668.

Muller, G.A., Hansen, U., Xu, Z., Griswold, B., Talan, M.I., McDonnell, N.B., and Briest, W. (2012). Allele-specific siRNA knockdown as a personalized treatment strategy for vascular Ehlers-Danlos syndrome in human fibroblasts. FASEB J *26*, 668-677.

Ohnishi, Y., Tokunaga, K., Kaneko, K., and Hohjoh, H. (2006). Assessment of allele-specific gene silencing by RNA interference with mutant and wild-type reporter alleles. J RNAi Gene Silencing *2*, 154-160.

Peek, A.S. (2007). Improving model predictions for RNA interference activities that use support vector machine regression by combining and filtering features. BMC Bioinformatics *8*, 182.

Ren, Y., Gong, W., Xu, Q., Zheng, X., Lin, D., Wang, Y., and Li, T. (2006). siRecords: an extensive database of mammalian siRNAs with efficacy ratings. Bioinformatics *22*, 1027-1028.

Schmidt, E.E., Pelz, O., Buhlmann, S., Kerr, G., Horn, T., and Boutros, M. (2013). GenomeRNAi: a database for cell-based and in vivo RNAi phenotypes, 2013 update. Nucleic Acids Res *41*, D1021-1026.

Shabalina, S.A., Spiridonov, A.N., and Ogurtsov, A.Y. (2006). Computational models with thermodynamic and composition features improve siRNA design. BMC Bioinformatics *7*, 65.

Thakur, N., Qureshi, A., and Kumar, M. (2012). VIRsiRNAdb: a curated database of experimentally validated viral siRNA/shRNA. Nucleic Acids Res *40*, D230-236.

Truss, M., Swat, M., Kielbasa, S.M., Schafer, R., Herzel, H., and Hagemeier, C. (2005). HuSiDa--the human siRNA database: an open-access database for published functional siRNA sequences and technical details of efficient transfer into recipient cells. Nucleic Acids Res *33*, D108-111.

Tyagi, A., Ahmed, F., Thakur, N., Sharma, A., Raghava, G.P., and Kumar, M. (2011). HIVsirDB: a database of HIV inhibiting siRNAs. PLoS One *6*, e25917.

Vert, J.P., Foveau, N., Lajaunie, C., and Vandenbrouck, Y. (2006). An accurate and interpretable model for siRNA efficacy prediction. BMC Bioinformatics *7*, 520.
